# Targeting the ARF6-dependent recycling pathway to alter lipid rafts and reduce inflammation

**DOI:** 10.1016/j.jlr.2025.100900

**Published:** 2025-09-16

**Authors:** Nigora Mukhamedova, Andrew J. Fleetwood, Kevin Huynh, Yangsong Xu, Tilly Van Buuren-Milne, Alexandra Faulkner, Ying Fu, Farhad Parhami, Peter J. Meikle, Ilya Levental, Michael Bukrinsky, Andrew J. Murphy, Dmitri Sviridov

**Affiliations:** 1 Baker Heart and Diabetes Institute, Melbourne, VIC, Australia; 2 MAX BioPharma Inc., Santa Monica, CA, USA; 3 Baker Department of Cardiovascular Research, Translation and Implementation, La Trobe University, Bundoora, VIC, Australia; 4 Department of Molecular Physiology and Biological Physics, University of Virginia, Charlottesville, VA, USA; 5 Department of Microbiology, Immunology and Tropical Diseases, George Washington University, Washington, DC, USA; 6 Department of Biochemistry and Molecular Biology, Monash University, Clayton, VIC, Australia

**Keywords:** Lipid raft, inflammation, cholesterol, oxysterol, ABCA1, membrane recycling, small GTPase

## Abstract

Partitioning of inflammatory receptors into lipid rafts is essential for their function making lipid rafts a promising target for anti-inflammatory therapies. However, the practical application of lipid raft-targeted therapies has been limited by a lack of mechanistic understanding of their regulation. In this study, we demonstrate that targeting the Arf6-dependent recycling pathway effectively modifies lipid rafts and mitigates inflammation. Using Oxy210, a synthetic oxysterol, we observed significant anti-inflammatory effects both in vivo and in vitro. These effects were linked to an increased abundance of ABCA1, enhanced cholesterol efflux, and alterations in the abundance and composition of lipid rafts. Mechanistically, Oxy210 disrupted the recycling checkpoint in late endosomes by reducing the abundance and activation of the small GTPase Arf6 and decreasing PI(4,5)P2 levels. This impaired lipid raft recycling to the cell surface and simultaneously reduced ABCA1 internalization. These findings suggest an approach to modulate lipid rafts, with potential applications in treating inflammation, neurodegeneration, and infectious diseases.

## Graphical Abstract

**Figure:**
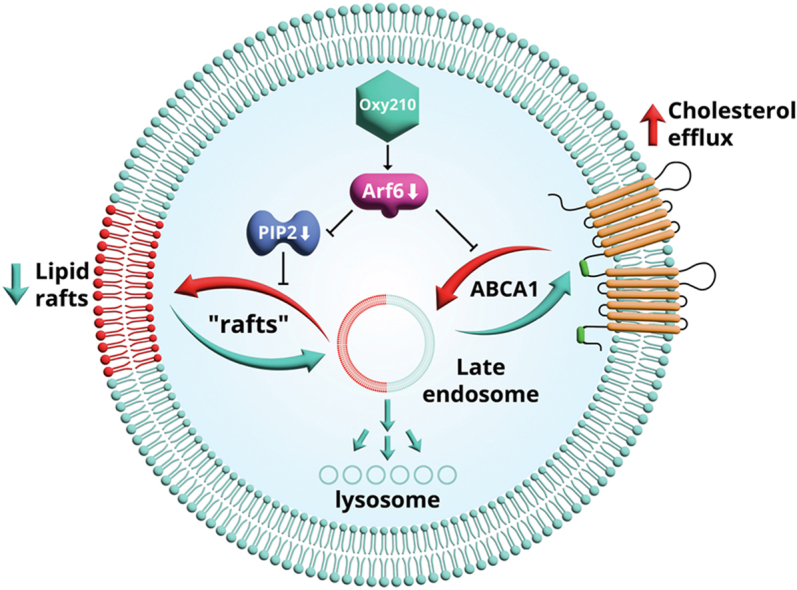

Lipid rafts are dynamic nanodomains within the plasma membrane, characterized by a liquid-ordered phase embedded in a liquid-disordered membrane environment (1, 2). Chemically, lipid rafts are distinguished by a high concentration of cholesterol, sphingomyelin, and saturated fatty acid chains, as well as a distinctive protein composition (3). Many glycosylphosphatidylinositol-anchored and lipid-modified proteins preferentially localize to these domains (4). Lipid rafts provide a favorable environment for structures that require close proximity of individual components to function, a condition that is difficult to maintain in a highly fluid membrane (5). This includes protein complexes whose activity depends on the rapid association/dissociation of their components (6), or on interactions with agonists or antagonists embedded in lipid rafts (7, 8). In addition, lipid rafts serve as platforms for receptors of various pathogens (9), transmembrane transporters (10), and amyloidogenic proteins (11).

Lipid rafts play a critical role in the pathogenesis of inflammation (12). These membrane domains accommodate receptors of the innate immune system, such as toll-like receptor 4 (TLR4) (13). TLR4 activation in response to ligand binding requires its dimerization, a process facilitated by lipid raft localization, which brings the two subunits into proximity (14). Notably, disruption of lipid rafts has been shown to inhibit TLR4-dependent inflammation (14, 15, 16). Beyond innate immunity, lipid rafts also harbor receptors involved in adaptive immune responses, and their disruption has been found to prevent B-cell and T-cell activation (6). In addition, lipid rafts play a critical role in regulating hematopoiesis and hematopoiesis-related inflammatory pathways (17).

Targeting lipid rafts to mitigate inflammation has been explored as a therapeutic strategy for various inflammatory conditions, including atherosclerosis (18), neuropathic pain (16), and neurodegenerative diseases (19). This highlights the potential of compounds capable of modulating the abundance or functional properties of lipid rafts as promising anti-inflammatory therapeutics. In this study, we investigate one such compound, the synthetic oxysterol Oxy210.

Oxysterols are generally considered inflammatory, atherogenic and harmful due to their enrichment in atherosclerotic plaques and the correlation between systemic levels of certain oxysterols and the severity of atherosclerosis (20). However, oxysterols represent a diverse group of compounds with varying biological effects. While some, such as 27-hydroxycholesterol, promote inflammation (21), others, like 25-hydroxycholesterol, exhibit anti-inflammatory properties (22, 23). Beyond their role in inflammation, oxysterols are key regulators of cholesterol metabolism (24, 25). The interplay between cholesterol metabolism and inflammation is well established, with lipid raft regulation serving as a critical link between these processes (26, 27).

In this study, we investigated the anti-inflammatory mechanisms of the synthetic oxysterol, Oxy210. The structure of Oxy210 has been previously reported (28) and is shown in supplemental Fig. S1. Our prior studies demonstrated that Oxy210 inhibits signaling from a variety of receptors (28, 29, 30, 31), most of which are known to localize within lipid rafts. Given that certain oxysterols can modify lipid raft properties (27, 32), we sought to determine whether Oxy210 exerts its effects by modulating abundance and/or functional properties of lipid rafts.

## Materials and Methods

### Animal studies

C57Bl/6J mice were housed under specific pathogen-free conditions on a standard 12/12 h light/dark cycle in the AMREP Animal Service Center. Food and water provided ad libitum.

Oxy210, formulated in 3% DMSO (Sigma-Aldrich) + 7% Ethanol (Sigma-Aldrich) + 5% PEG400 (Sigma-Aldrich) + 85% corn oil (Sigma-Aldrich), was administered to the mice once daily for 4 days (*ie* 4 doses) by oral gavage at a dose of 200 mg/kg (33). The vehicle control group was administered the formulation solution alone. Two hours after the final oral gavage, a subset of the mice was injected intraperitoneally with lipopolysaccharide (LPS) (5 μg/mouse, *Escherichia coli*; Invivogen) in 200 μl of PBS; mice were humanely euthanized 2 h after the LPS injection. Blood, bone marrow (BM), and liver were collected for flow cytometry and plasma collected for cytokine and lipid analysis. Peritoneal cells were harvested by injecting 4 ml of ice-cold PBS into the peritoneal cavity; after gentle massaging, the fluid was aspirated and the cells collected by centrifugation (500 g for 5 min). Livers were dissected from mice and immediately placed on ice. Livers were homogenized and flushed through a 40 μm cell strainer with PBS to obtain a single cell suspension. Red blood cells (RBCs) were lysed, and cells resuspended in HBSS buffer.

Total white blood cell (WBC) count was obtained from freshly collected blood or bone marrow diluted in cell pack buffer (1:7) using the Sysmex XN-550i automated hematology analyzer (capillary mode).

All animal experiments were approved by the Animal Ethics Committee of the Alfred Research Alliance (approval no. P8443) and conducted in accordance with the Australian Code of Practice for the Care and Use of Animals for Scientific Purposes as stipulated by the National Health and Medical Research Council of Australia. The mice were randomly assigned to treatment.

### Cells

Bone marrow-derived macrophages (BMDMs) were isolated from tibia and femur bones of 6–8 week-old C57Bl/6J male mice as described by Shrestha *et al.* (34). In brief, bone marrow was flushed out from bones with Iscove's modified Dulbecco's medium (IMDM) containing 5% fetal bovine serum (FBS, Bovogen). Cells were spun, pellet washed and incubated for 10 min in red blood cell lysis buffer (155 mM NH_4_Cl, 10 mM KHCO_3_, 0.1 mM EDTA, pH 7.3). Cell pellet was resuspended in IMDM containing 5% FBS, spun once more and resuspended in IMDM containing 10% FBS and 15% of L929 cell conditioned media (35). Cells were plated in Petri dishes (BD) and cultured for 7 days.

Mouse macrophage cells RAW264.7 (ATCC, Manassas, VA) were grown in RPMI-1640 medium supplemented with 10% FBS. Human T lymphoid cells SUP-T1 (ATCC) were maintained in RPMI-1640 medium supplemented with 10% FBS. Human neuroblastoma cells SH-SY5Y (ATCC) were maintained in DMEM/F12 medium containing 10% FBS. For experiments, cells between passages 13 and 25 were seeded onto rat collagen 1-coated plates and underwent primary differentiation by a 5-day incubation with complete media supplemented with 10 μM retinoic acid (Sigma–Aldrich, #R2625), followed by a secondary differentiation. Cells were incubated for 2 days in serum-free DMEM/F12 medium containing brain-derived neurotrophic factor (BDNF) (Abcam, #9794) at 5 ng/ml. The cells were maintained in serum-free brain-derived neurotrophic factor-containing media. Cell differentiation was confirmed visually by morphologic changes and spread of neurites as well by staining for MAP2. The human skin fibroblasts from a patient with Tangier disease as well as normal skin fibroblasts were a kind gift of Dr A. Remaley (NHLBI/NIH). The cells were grown in DMEM containing 10% FBS and 100 U/ml Pen-Strep.

### Lipid raft isolation and analysis

Cells were labeled by incubation in serum-containing medium with 7.5 kBq/ml of [1a,2a (n)-^3^H]cholesterol] (GE Healthcare) added in ethanol for 48 h and thoroughly washed with PBS. Cells were incubated for 18 h in medium containing 0.1% FBS in the presence of 5 μMol/L Oxy210. Lipid rafts were isolated from a post nuclear supernatant by flotation through a continuous OptiPrep (STEMCELL, #07820) density gradient centrifugation as described by Mukhamedova *et al.* (36). In brief, cells were cooled on ice, washed with PBS, collected, and resuspended in 5 mM Tris–HCl buffer (pH 7.5) supplemented with a mixture of protease/phosphatase inhibitors (Pierce, #A32959). Hypoosmotic shock combined with repeated freezing-thawing was used to prepare post nuclear fraction. After spinning down cellular debris at 300 g for 5 min at 4°C; supernatant was collected and centrifuged at 45,000 g 90 min at 4°C. The pellet from this centrifugation was resuspended in 23% iodixanol solution containing protease/phosphatase inhibitors, loaded in tube containing OptiPrep 10%–20% linear gradient and centrifuged in swing-bucket rotor for 90 min at 52,000 g). The top forty percent of the tube content was transferred to another tube. Fifteen and 5% of iodixanol were layered over the samples and centrifuged at 52,000 g for 90 min. Fifty microliters fractions were collected from the top and analyzed either for radioactivity using β-counter or flotillin-1 abundance by western blotting.

### Flow cytometry

#### Blood leukocytes

Monocytes (total and subsets), neutrophils, T cells, and B cells were identified from whole blood as previously described (37). Blood was collected by cardiac puncture into EDTA lined tubes and immediately placed on ice. All following steps were performed on ice. RBCs were lysed and WBCs were centrifuged, washed, and resuspended in fluorescence-activated cell sorting (FACS) buffer (Hanks balanced salt solution + 0.1% BSA w/v, 5 mM EDTA). Cells were stained with a mixture of antibodies against CD45 (BD Biosciences, #564279), Ly6-C/G (Gr-1) (Invitrogen, #108428), CD115 (BioLegend, #135524), CD3 (BioLegend, #100204), and B220 (BioLegend, #103206). Monocytes were identified as CD45^+^CD115^hi^ and further identified into Ly6-C^hi^ and Ly6-C^lo^; neutrophils were identified as CD45^+^CD115^lo^Ly6-C/G^hi^ (Gr-1). T cells were identified as CD45^+^, CD115^−^, Gr1^−^, CD3^+^, and B220^−^. B cells were identified as CD45^+^, CD115^−^, Gr1^−^, CD3^−^, and B220^+^. TLR4 (BioLegend, #117602, #145409) binding in the stated subsets was also measured. All antibody staining was done at 1:400 dilution for 30 min in the dark on ice in FACS buffer.

#### Peritoneal macrophages

Peritoneal cell suspensions were incubated for 30 min with a cocktail of antibodies against CD45, CD11b (Invitrogen #MA1-10081), CD11c (BioLegend#117324), F4/80 (BioLegend, #111604), and CD19 (BioLegend, #152416). Longer-lived peritoneal macrophages (LPM; CD45^+^CD19^−^CD11c^−^CD11b^+^F4/80^+^FSC-A^hi^) and shorter-lived peritoneal macrophages (SPM; CD45^+^CD19^−^CD11c^−^CD11b^mid^F4/80^mid^FSC-A^low^) were characterized (38). TLR4 binding in the LPM and SPM was also measured. All antibody staining was done at 1:400 dilution in the dark on ice in FACS buffer.

#### Kupffer cells

Liver cell suspensions were incubated for 30 min with a mixture of antibodies against CD45, CD11b, F4/80, CD64 (BioLegend, #139308), MHCII (BioLegend, #1107645), and TIM4 (BioLegend, #1130022). Kupffer cells were characterized as described (39). All samples were stained with viability stain (BD Biosciences, #565388, 1:2000).

#### Hematopoietic stem and progenitor cells

Hematopoietic stem and progenitor cells from the BM were analyzed by flow cytometry as previously described (40). BM was harvested from femurs and tibias by flushing with ice cold PBS. RBCs were lysed and WBCs were centrifuged, washed, and resuspended in FACS buffer. Single cell suspensions were stained with a cocktail of antibodies against lineage-committed cells (B220, CD19, CD11b, CD3e, and TER-119) (BioLegend, #116206), Sca1 (BioLegend, #122520), c-Kit (BioLegend, #105826), Flt3 (BioLegend,#135306), CD48 (BD Biosciences, #740236), CD150 (BioLegend, #115927), CD16/CD32 (BD Biosciences, #740217), and CD34 (BioLegend,#128610). LSK subsets were characterized as LT-HSC (CD48^−^CD150^+^LSK), ST-HSC (CD48^−^CD150^−^LSK), MPP2 (CD48^+^CD150 ^+^LSK), MPP3 (CD48^+^CD150^−^LSK), and MPP4 (CD48^+^Flt3^+^ CD150^−^LSK). HSPC subsets were characterized as myeloid-erythroid progenitor cells (CD16/32^−^CD34^+^cKit^+^), CMP (CD16/32^int^CD34^int^cKit^+^), and GMP (CD16/32^+^CD34^+^cKit^+^). All samples were stained with viability stain (BD Biosciences, #565388). All antibody staining was done at 1:400 dilution for 40 min in the dark on ice in FACS buffer. Staining was stopped with FACS buffer and cells were subsequently washed and filtered through a 35-μm strainer before analysis. FACS was performed at the ARA Flow Cytometry Core Facility on a LSRII Fortessa (BD Biosciences). Data were analyzed using FlowJo software v.10.9 (BD).

### Cytokine secretion measurements

Tumor necrosis factor α (TNFα) and interleukin 6 (IL-6) in mouse plasma and in the cell culture media were measured by commercial ELISA (BioLegend, #430904, #431304) according to manufacturer’s protocol using Varioskan Lux Plate Reader (Thermo Fisher Scientific) or Benchmark Plus (Bio-Rad). For in vitro experiments, BMDMs were stimulated for 4 h with 100 ng/ml LPS, treated with indicated concentrations of Oxy210 for 24 h. Cell culture medium was collected, and cytokine levels were measured. Cells were lysed and protein concentration measured by Bradford assay (Bio-Rad). Final cytokine concentration was normalized to cellular protein content.

### TLR4 dimerization assay

TLR4 dimerization and endocytosis were assessed essentially as before (41). Briefly, BMDMs were treated with Oxy210 or vehicle control ± LPS (1 μg/ml) for 15 min at 37°C. Untreated control samples were also collected at 15 min. BMDMs were washed twice with cold PBS and stained with anti-TLR4 antibodies to assess dimerization (TLR4, clone MTS510, BioLegend #117602) and endocytosis (TLR4, Clone SA15-21, BioLegend, #145409) for 30 min on ice. BMDMs were also stained for cholera toxin B (CtxB-AF488, 1:2000) for 30 min on ice. Stained cells were washed with 1 ml FACS buffer, resuspended in 150 μl of FACS buffer, and surface staining analyzed on a LSRII Fortessa (BD Biosciences). The mean fluorescence intensity (MFI) of TLR4 was recorded and the percentage dimerization and endocytosis of TLR4 calculated as described (42). Briefly, the percentage of the TLR4 monomer was determined by the ratio of the MFI values (obtained from MTS510 antibody staining) of the LPS or Oxy210 stimulated cells to those of the vehicle control treated cells. For measuring the extent of TLR4 dimerization, the percentage of TLR4 dimer was calculated as the percentage of TLR4/MD-2 monomer.

### Cholesterol efflux

Human high density lipoprotein (HDL) and apolipoprotein A-I (apoA-I) were isolated from pooled human plasma as described by Morrison *et al.* (43).

Cholesterol efflux assay has been described previously (44). In brief, cells were labeled by incubation in serum-containing medium supplemented with [1a,2a (n)-^3^H] cholesterol added in ethanol at the final activity of 75 kBq/ml for 48 h. Cells were washed, incubated for 18 h in medium containing 0.1% FBS in the presence or absence of 5 μMol/L Oxy210, and washed again. Serum-free media without (blank) or with acceptor (either human apoA-I added at final concentration of 20 μg/ml or human HDL final concentration of 30 μg/ml) was added and cells were incubated for 2 h at 37°C. The efflux was expressed as a proportion of radioactivity moved from cells to medium minus nonspecific efflux (ie the efflux to the medium without acceptor).

### Cholesterol biosynthesis

Cholesterol biosynthesis was assessed as described previously (45). BMDMs were seeded in IMDM containing 10% FBS and incubated for 24 h, washed and treated with 5 μMol/L Oxy210 in IMDM containing 0.1% FBS for 18 h. Cells were then washed and incubated for 4 h at 37°C in serum-free IMDM containing 0.1% BSA and [^3^H]acetate (final radioactivity of 7.4 MBq/ml). Cells were washed twice and collected in distilled water. Lipid extraction and TLC using cholesterol and cholesteryl estres system was done as described previously (46).

### Western blotting

Cells were lysed with RIPA buffer, protein concentration in lysates estimated by Bradford assay followed by SDS-PAGE and transfer of proteins to PVDF membrane. Semiquantitative analysis of western blots was performed by densitometry and presented as a proportion of control after normalization to loading controls. Antibodies used in western blot analysis:ATP binding cassette transporter A1 (ABCA1) (Abcam, #ab18180, 1:3000), ATPase (Abcam, #ab76671, 1:5000), GAPDH (Merck, #CB1001, 1:5000), ARF GTPase 6 (Arf6) (Sigma-Aldrich, #A5230, 1:2000), cell division cycle control protein 42 (Cdc42) (BD transduction Laboratories, #610929, 1:500), mitogen-activated protein kinase 1/2 (Erk1/2) (Millipore, #66192, 1:10,000), pErk1/2 (Cell Signaling Technology, #4370, 1:2000), TMS1(Cell Signaling Technology, #67824 1:1000), NRLP3 (Cell Signaling Technology, #15101, 1:1000), Caspase-1 ((Cell Signaling Technology, #24232, 1:1000), ATP binding cassette transporter G1 (ABCG1) (Novus Biologicals, #NB400-132, 1:1000), Anti-mouse HRP-linked antibody (Cell Signaling Technology, #7076, 1:5000), Anti-rabbit HRP-linked antibody (Cell Signaling Technology, #7074, 1:5000).

### Transfections

To silence *Cdc42* or *Arf6* in RAW264.7 cells On -TARGETplus control siRNA (#D-001810), On -TARGETplus *Cdc42* siRNA (#J-043087-09) or On -TARGETplus *Arf6* siRNA (#J-043217-09) Dharmacon) were transfected into cells using Lipofectamine RNAiMAX (Invitrogen, #13778030)) according to manufacture protocol.

To transfect RAW264.7 cells with control pCMV, pcDNA3 HA *Arf6* ActQ67L, or pcDNA3 HA *Arf6* DN T27N (Addgene) Lipofectamine LTX with Plus reagent (Invitrogen, #15338100) was used according to manufacturer protocol.

### Real time PCR

BMDMs seeded in 6-well plates and treated with either 5 μMol/L Oxy210 or 4 μMol/L TO-901317 for 18 h were collected in cold PBS, and total RNA was extracted using PureLink RNA Mini Kit (Invitrogen, 12183018A) according to the manufacturer’s protocol. Complementary DNA was synthesized from 2 μg of RNA with random primers using the M-MLV Reverse Transcriptase kit (Promega, M1701).

Real Time PCR was performed in triplicates using Taqman Fast Advanced PCR Master mix (Applied Biosystems, #4444557) with the following primers:- Abca1 (Mm00442646_m1); Abcg1 (Mm00437390_m1); ApoE (Mm01307193_g1); Srebf (Mm01306292_m1); Scarb1 (Mm00450234_m1); Dnm2 (Mm00514582_m1), Il6 (Mm00446190_m1), Tnf (Mm0044258_m1). All Taqman primers were from Applied Biosystems. Real-time PCR reactions were performed on a 7,500 Fast System from ABI Applied Biosystems. The relative amount of mRNA was calculated with the comparative C_T_ method. Gene expression was normalized to 18 s rRNA.

### Lipidomic analysis

#### General lipidomic analysis

Lipidomic analysis was performed as described previously (47). Combined lipid raft fractions or cell lysate were sonicated at 0°C; lipids were extracted using a modified single phase 1:1 butanol:methanol method (48). In brief, samples were lyophilized and mixed with 50 μl of methanol, 50 μl of butanol, and 10 μl of water. Samples were sonicated for 1 h at 25°C before centrifugation (13,000 g) for 10 min. The supernatant was carefully aspirated, avoiding the optiprep precipitate, into mass spectrometry glass vials with inserts.

Lipids were separated under the following chromatographic conditions using solvent A (50% water, 30% acetonitrile, and 20% isopropanol with 10 mM ammonium formate and 5 μM medronic acid) and solvent B (1% water, 9% acetonitrile, and 90% isopropanol with 10 mM ammonium formate). Starting at 15% B, going to 50% at 2.5 min, 57% at 2.6 min, 70% at 9 min, 93% at 9.1 min, 96% at 11 min, 100% at 11.1 min, and holding until 12 min before going down to 15% at 12.2 min and re-equilibrating at 15% B until 16 min.

Mass spectrometer (Agilent 6495C) conditions for fractions were as follows: gas temperature, 200°C; gas flow rate, 17 L/min; nebulizer, 20 psi; sheath gas temperature, 325°C; capillary voltage, 3500 V; and sheath gas flow, 10 L/min. Relative lipid concentrations for each fraction was calculated by relating the peak area of each species to the peak area of the corresponding internal standard.

#### Oxy210 measurement

Samples were run under a separate chromatographic gradient to measure Oxy210. Starting at 0% B and going to 50% B at 6 min, 100% B at 8 min and holding until 10 min, before going back to 0% at 11 min and re-equilibrating until 14 min. Oxy210 was measured with a precursor ion of 424.6 [M+H]^+^ with the product ions 226.2 and 406.3 were qualifier ions with 159.1 as the quantifier ion. Relative abundances were estimated using the cholesterol-d7 internal standard.

To measure the distribution of Oxy210 in murine plasma, VLDL/LDL (1.006 < d < 1.063 g/ml), HDL (1.063 < d < 1.21 g/ml), and lipoprotein-deficient plasma (LPDP) (d > 1.21 g/ml) were isolated by sequential ultracentrifugation in solutions of KBr of appropriate density as described by Havel *et al.* (49). Concentration of Oxy210 and Oxy210 esters in plasma fractions was determined as described above and related to total cholesterol level (cholesterol and cholesteryl esters).

#### Phosphatidylinositol phosphates

BMDM pellets were lyophilized overnight before extraction. Lipids were extracted in Eppendorf tubes using a single-phase extraction method with 2:2:1 butanol:methanol:water with 200 mM of ammonium formate and 1 mM of medronic acid. 500 pmol of phosphatidylinositol phosphate (PIP) internal standards, rac-16:0PI(3,5)P2-d5 (Avanti) and rac-16:0 PI(5)P-d5 (Avanti), was also added into each sample. Samples were vortexed and sonicated on a sonicator bath for 1 h before centrifugation at 11,000 g for 10 min. The supernatant was transferred into glass vials for mass spectrometry analysis.

PIPs were measured under identical chromatographic conditions except using a BEH Premier C18 column (2.1 x 100 mm, 1.7 μm, Waters) operated at 45^°^C. Mass spectrometer (Agilent 6495C) conditions for PIPs were as follows: gas temperature, 230°C; gas flow rate, 13 L/min; nebulizer, 15 psi; sheath gas temperature, 350°C; capillary voltage, 4,000 V; and sheath gas flow, 12 L/min. Species in phosphatidylinositol monophosphate and diphosphate classes were measured in positive mode as [M+H]^+^ ions with the neutral loss of the phospho-inositol and the additional phosphates as the product ion. Relative concentrations were calculated by normalizing the species against the corresponding internal standards.

HDL cholesterol was measured by colorimetric assay (FUJIFILM Waco, #299-96501) according to the manufacturer instructions.

#### Arf6 activation assay

Arf6 activation pull-down assay (Cytoskeleton, #BK033-S)) was implemented according to the manufacturer’s protocol. Briefly, RAW264.7 cells were seeded at 40% confluency on Petri dishes. The next day cells were washed with PBS and left in RPMI containing 0.1% FBS with or without 5 μMol/L Oxy210 for 18 h. Cells were washed with ice-cold PBS, lysed, centrifuged (2 min 10,000 g) and the supernatant was snap-frozen. Four hundred micrograms of total protein was incubated with GGA3-PBD Sepharose beads with affinity to activated Arf6. After washing with PBS, beads were incubated with Laemmli buffer then boiled and separated with spin columns. Eluates were subjected to SDS-PAGE and western blot with anti-Arf6 antibody.

#### Protein lipid overlay assay

Binding of Arf6 to Oxy210 was assessed using the protein lipid overlay assay as described by Dowler *et al.* (50). In brief, indicated amounts of Oxy210 were applied on a nitrocellulose membrane (2 μl/dot). Vehicle was added as negative controls, and recombinant Arf6 (0.2 μg) (Abcam, #ab102025) was added as a detection control. The membrane was dried for 1 h and then incubated for 1 h in the blocking solution (TBS containing 0.1% Tween and 3% fatty acid free BSA) at room temperature. The membrane was then incubated overnight at 4°C with recombinant human Arf6 (1 μg/ml), washed, incubated for 2 h at room temperature with anti-His Tag monoclonal antibody (1:1,000), washed and incubated for 1 h at room temperature with secondary polyclonal HRP-conjugated antibody (1/4,000).

#### Statistics

Statistical analyses were performed with GraphPad Prism and MS Excel. Unpaired two-tail *t*-test, ANOVA or Mann-Whitney test were used as indicated. Details on the number of biological replicates and *P* values are provided in the corresponding figure/table legends. Nonsignificant (NS) implies *P* > 0.05.

## Results

### Oxy210 reduces inflammation

**Fig. 1:**
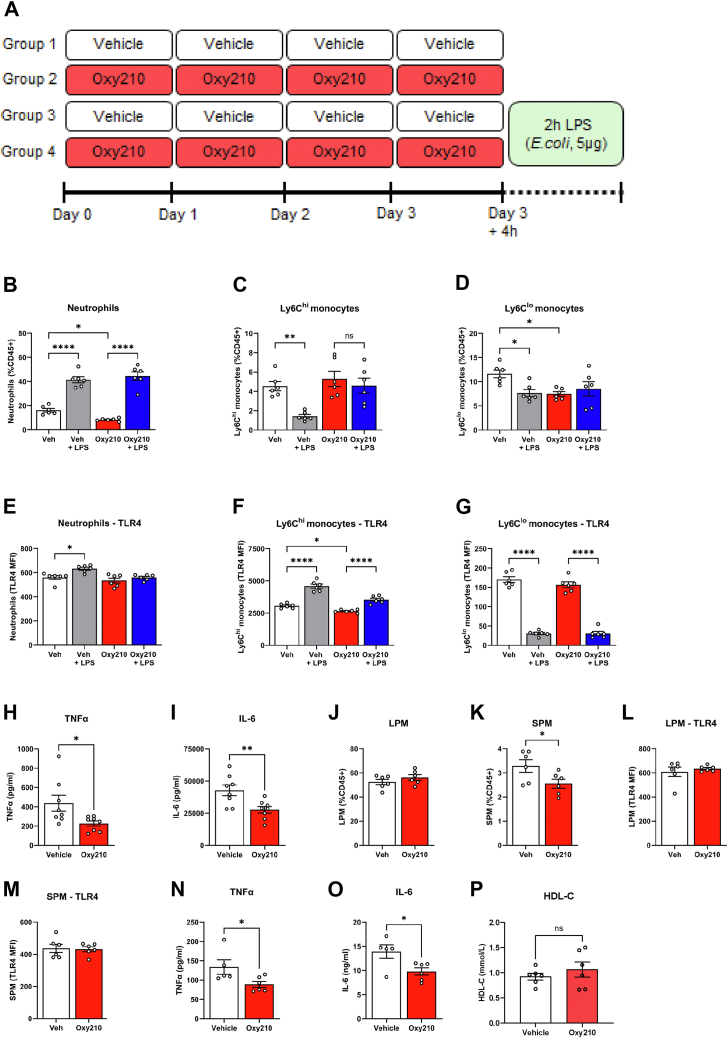
The effect of Oxy210 on inflammation in vivo. A**:** Schematic diagram of the experimental design. B–D: Cell frequency (% of CD45) of neutrophils (B), Ly6C^hi^ monocytes (C), and Ly6^lo^ monocytes (D) in the blood. E-G: TLR4 abundance (MFI) on the surface of neutrophils (E), Ly6C^hi^ monocytes (F), and Ly6^lo^ monocytes (G) in the blood. H and I: Plasma levels of TNFα (H) and IL-6 (I) from vehicle control or Oxy210-treated animals challenged with LPS. J and K: Cell frequency of large (LPM) (J) and small (SPM) (K) peritoneal macrophages in vehicle control or Oxy210 treated animals. L and M: TLR4 abundance (MFI) on the surface of LPM (L) and SPM (M) cells. N and O: Secretion of TNFα (N) and IL-6 (O) from peritoneal macrophages isolated from vehicle control or Oxy210-treated animals and stimulated ex vivo with LPS (2 h). P: Plasma level of HDL cholesterol measured by colorimetric assay. Mean ± SEM are shown. ∗*P* < 0.05; ∗∗*P* < 0.01; ∗∗∗*P* < 0.001; ∗∗∗∗*P* < 0.0001 versus vehicle (ANOVA). LPS, lipopolysaccharide; LPM, longer-lived peritoneal macrophages; SPM, shorter-lived peritoneal macrophages; TNFα, tumor necrosis factor α; IL-6, interleukin 6; MFI, mean fluorescence intensity; TLR, toll-like receptor.

To further investigate the effect of Oxy210 on inflammation, mice were administered Oxy210 by oral gavage (200 mg/kg) once daily over 4 days; control mice were fed with the same volume of vehicle (corn oil) (Fig. 1A). At the end of the feeding period, a subset of mice in each group was injected intraperitoneally with LPS (5 μg per mouse); 2 h later the experiment was terminated.

In the blood, treatment of mice with Oxy210 reduced the frequency of circulating neutrophils and Ly6C^lo^ (patrolling) monocytes, while Ly6C^hi^ (inflammatory) monocytes were not affected (Fig. 1B–D). Induction of inflammation by intraperitoneal LPS injection led to an elevation in circulating neutrophils, which was not influenced by Oxy210. Following a peritoneal challenge with LPS, Ly6C^hi^ monocytes are rapidly recruited from the blood to the inflamed peritoneum (51). Consistent with this, the frequency of circulating Ly6C^hi^ monocytes decreased in control mice whereas their frequency was unchanged in Oxy210-treated animals. Treatment with LPS increased the abundance of TLR4 on the surface of neutrophils and Ly6C^hi^ monocytes, Oxy210 effectively prevented this elevation (Fig. 1E–G). Finally, after LPS injection, the concentrations of TNFα and IL-6 in plasma of Oxy210-treated animals were reduced (Fig. 1H, I).

We then used peritoneal lavage to assess the abundance and properties of myeloid cells in the peritoneum. Although the frequency of so-called large peritoneal macrophages (LPM) was unaffected by Oxy210, the frequency of the rarer SPM (short-lived Ly6C^hi^-derived cells) was reduced in animals treated with Oxy210 (Fig. 1 J, K). We did not find significant changes in TLR4 abundance on the surface of peritoneal macrophages due to treatment with Oxy210 (Fig. 1L, M). When peritoneal macrophages isolated from animals not treated with LPS were plated and treated with LPS ex vivo, secretion of TNFα and IL-6 was reduced in macrophages from Oxy210-treated animals (Fig. 1N, O).

We also analyzed bone marrow cells from animals treated or not with Oxy210. Oxy210 had no effect on the frequency of various hematopoietic stem cell populations (supplemental Fig. S1A–F), myeloid progenitor cells (supplemental Fig. S2A–C), or bone marrow leukocytes (supplemental Fig. S2D–G), except for a modest increase in myeloid–erythroid progenitor cells in Oxy210+LPS-treated animals (supplemental Fig. S2C). In addition, Oxy210 did not alter surface TLR4 abundance in BM cells (supplemental Fig. S4H–L). Analysis of Kupffer cells isolated from the liver also showed no changes in cell frequency or TLR4 expression following Oxy210 treatment (supplemental Fig. S2M, N). Thus, while Oxy210 reduced monocyte frequency and inhibited LPS-induced inflammation in blood and peritoneal monocytes, it did not affect hematopoietic or liver cells.

Lipid composition of plasma was assessed by lipidomics analysis and is shown in Table 1. Oxy210 was found in plasma of animals fed with Oxy210 but was absent in plasma of control animals; 85% of Oxy210 was in free form and 15% was esterified. Oxy210 had no effect on plasma total or esterified cholesterol content or triglycerides. Treatment of mice with LPS resulted in a surprising 80% reduction of Oxy210 levels in plasma, the reasons for that are unclear. LPS increased plasma triglyceride level, but Oxy210 reduced it to a level below that in control mice. Inflammation-induced hypertriglyceridemia is an established phenomenon (52) and reduction of plasma triglyceride levels may be a reflection of an anti-inflammatory property of Oxy210. Plasma levels of total cholesterol and cholesteryl esters after treatment with LPS were unaffected by Oxy210. Plasma HDL cholesterol levels were assessed by colorimetric method and were not affected by Oxy210 (Fig. 1P)).

**Table 1:** The effect of Oxy210 on concentrations of plasma lipids

Treatment	Oxy210 (mmol/L)<	Cholesterol (mmol/L)	Cholesteryl Esters (mmol/L)	Triglycerides (mmol/L)
Vehicle	0	2.7 ± 0.3	1.9 ± 0.3	1.2 ± 0.3
Vehicle + LPS	0	2.5 ± 0.5	1.8 ± 0.4	3.6 ± 0.7∗∗
Oxy210	0.028 ± 0.014	2.6 ± 0.5	1.8 ± 0.4	1.4 ± 0.4
Oxy210+ LPS	0.0006 ± 0.0005	2.9 ± 0.2	2.1 ± 0.2	0.7 ± 0.2∗

LPC, lipopolysaccharide.

Plasma lipids were measured by mass spectrometry (Lipidomics). Mean ± SEM are shown; *P* < 0.01; *P* < 0.001; (versus vehicle, *t*-test, n = 6).

To examine the distribution Oxy210 among plasma lipoproteins, we isolated lipoproteins from plasma samples of mice treated with Oxy210 as described above (Fig. 1A, group 2) and measured concentration of Oxy210 and its esters in isolated lipoproteins and LPDP by mass spectrometry. We found 57% of Oxy210 residing in LPDP, 37% in HDL, and remaining 6% in LDL/VLDL fractions (Table 2). Eighty percent of Oxy210 esters were found in the HDL fraction and remaining 20% split between VLDL/LDL and LPDP fractions. Thus, Oxy210, especially its esters, have a preference of binding to HDL, however, majority of unesterified Oxy210 is not carried by lipoproteins, consistent with more polar structure comparing to cholesterol (supplemental Fig. S1).

**Table 2:** Distribution of Oxy210 in plasma

Compound	LDL/VLDL		HDL		LPDP
	(mmol/mol TC)	% of total	(mmol/mol TC)	% of Total	% of Total
Oxy210	0.6 ± 0.1	5.9 ± 1.9	0.9 ± 0.3	37.1 ± 8.6	57.0 ± 5.8
Oxy210 esters	0.5 ± 0.1	8.5 ± 1.3	0.9 ± 0.1	80.6 ± 22.6	10.9 ± 2.1

LPDP, lipoprotein-deficient plasma.

Mice were administered Oxy210 by oral gavage (200 mg/kg) once daily over 4 days. Plasma samples were taken upon termination of the experiment and lipoproteins isolated by sequential ultracentrifugation in KBr solutions of different densities as described in the “Materials”. Concentration of lipids was measured by mass spectrometry (Lipidomics). Percentages were calculated relative to the total amount of Oxy210 in all lipoprotein fractions and LPDP. Means ± SEM are shown, n = 3.

### Oxy210 reduces abundance of lipid rafts

**Fig. 2:**
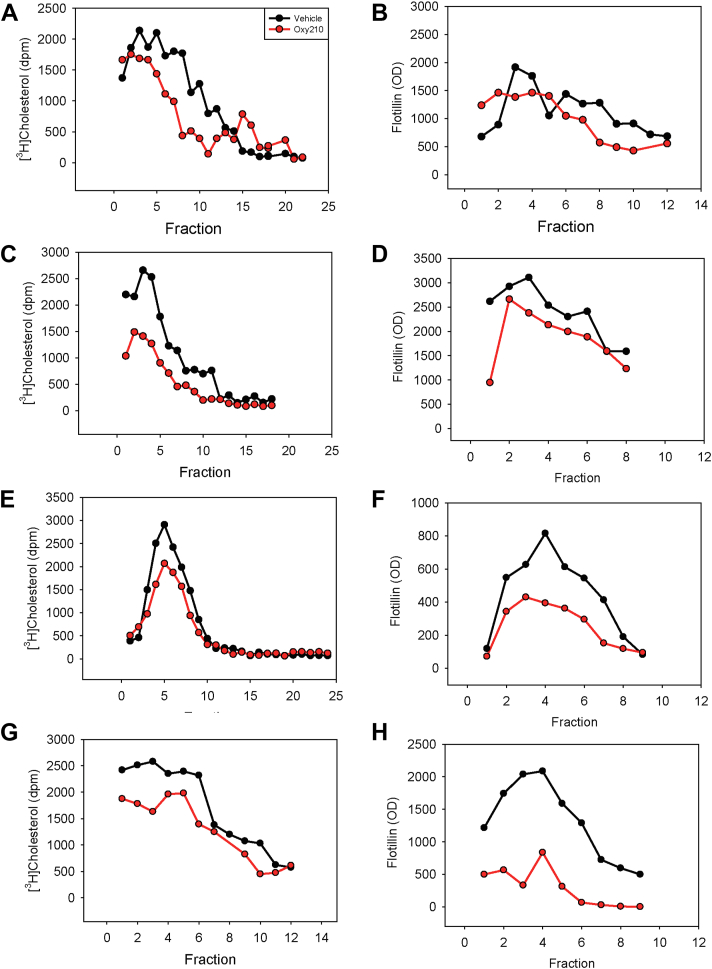
The effect of Oxy210 on the abundance of lipid rafts in various cell types. Cells were labeled with [^3^H]cholesterol and isolated plasma membranes were fractionated by density gradient centrifugation as described in “Methods”. Lipid rafts were defined as fractions with highest [^3^H] cholesterol and flotillin content. Area under the curve values and statistics are shown in Table S1. A and B: The effect of Oxy210 (5 μM) on lipid raft abundance in SupT1 cells. Distribution of [^3^H] cholesterol (A) and flotillin-1 (B) in the density gradient fractionation of plasma membrane. C and D: The effect of Oxy210 (5 μM) on lipid raft abundance in RAW264.7 cells. Distribution of [^3^H] cholesterol (C) and flotillin-1 (D) in the density gradient fractionation of plasma membrane. E and F: The effect of Oxy210 (5 μM) on lipid raft abundance in BMDMs. Distribution of [^3^H]cholesterol (E) and flotillin-1 (F) in the density gradient fractionation of plasma membrane. G and H: The effect of Oxy210 (5 μM) on lipid raft abundance in SH-SY5Y cells. Distribution of [^3^H] cholesterol (G) and flotillin-1 (H) in the density gradient fractionation of plasma membrane. For panels A, C, E, and G - *P* < 0.05 (Mann-Whitney test). BMDM, bone marrow-derived macrophage.

We hypothesized that the anti-inflammatory activity of Oxy210 could be explained by its capacity to reduce the abundance of lipid rafts. To assess the effect of Oxy210 on the abundance of lipid rafts in various cells, we isolated and quantitated lipid rafts by assessing the abundance of cholesterol and lipid raft marker flotillin-1 as described previously (36, 53). Cells were labeled with [^3^H] cholesterol and equal amounts of post nuclear supernatant were fractionated by density gradient centrifugation. Profiles of [^3^H] cholesterol and flotillin-1 distribution along the density gradient are shown in Figure 2, and area under the curve values and statistics are shown in supplemental Table S1. Light fractions containing elevated amounts of [^3^H] cholesterol and enriched in flotillin-1 (typically, fractions 2–9) were defined as lipid raft fractions. Treatment of cells with Oxy210 at 5 μmol/L for 18 h resulted in a statistically significant reduction in the abundance of lipid rafts in SupT1 human lymphoma cells (Fig. 2A, B), RAW264.7 murine macrophages (Fig. 2C, D), murine BMDMs (Fig. 2E, F) and SH-SY5Y human neuroblastoma cells (Fig. 2G, H). There was a strong correlation between lipid raft quantification based on determination of cholesterol or flotillin-1 abundance. The observed effects were consistent across all studied cell types. Thus, Oxy210 reduces the abundance of lipid rafts in various cells.

### Oxy210 reduces TLR4 signaling

**Fig. 3:**
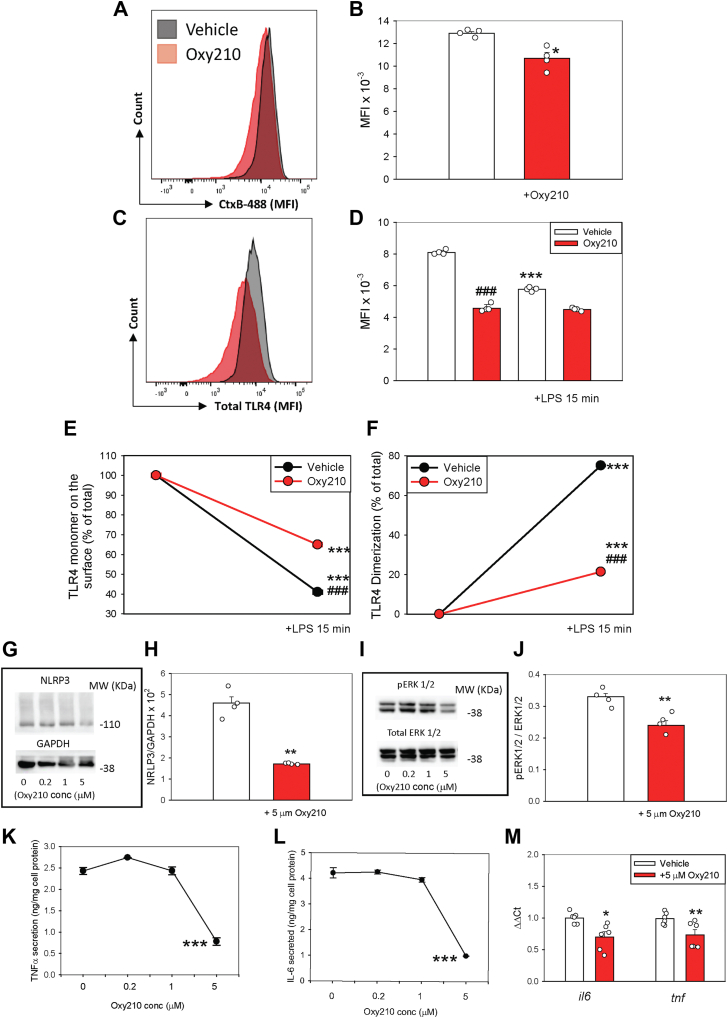
The effect of Oxy210 on inflammation in vitro. A and B: The effect of Oxy210 (5 μM) on the abundance of lipid rafts in BMDM as measured by flow cytometry. Representative FACS plots for CTxB levels (A) and mean fluorescence intensity (MFI) of CTxB (B) on BMDM. N = 4; ∗*P* < 0.05 versus vehicle (*t*-test). C and D: The effect of Oxy210 (5 μM) and LPS (1.0 μg/ml for 15 min) on the abundance of TLR4 on the surface of BMDM as measured by flow cytometry. Representative FACS plots for TLR4 levels (C) and the MFI of TLR4 (D) on BMDM. N = 4; ∗∗∗*P* < 0.001 versus “no LPS”; ^###^*P* < 0.001 versus vehicle (*t*-test). E: The effect of Oxy210 (5 μM) on the abundance of TLR monomer on the surface of BMDM measured by flow cytometry as described in “Methods”. The graph shows changes in the abundance of TLR4 monomer after treatment with LPS (1.0 μg/ml for 15 min; relative to its abundance before the treatment). N = 4; ∗∗∗*P* < 0.001 versus “no LPS”; ^###^*P* < 0.001 versus vehicle (*t*-test). F: The effect of Oxy210 (5 μM) on the proportion of TLR dimer on the surface of BMDM measured by flow cytometry as described in “Methods”. The graph shows changes in the abundance of TLR4 dimer after treatment with LPS (1.0 μg/ml for 15 min; relative to its abundance before the treatment). N = 4; ∗∗∗*P* < 0.001 versus “no LPS”; ^###^*P* < 0.001 versus vehicle (*t*-test). G: The effect of different concentrations of Oxy210 on the abundance of NLRP3 in BMDM in response to treatment with LPS for 4 h. H: The effect of Oxy210 (5 μM) on the abundance of NLRP3 in BMDM in response to treatment with LPS. Densitometry of western blots (n = 4, biological replicates, ∗∗*P* < 0.001, *t*-test). I: The effect of different concentrations of Oxy210 on the abundance of total and phosphorylated ERK1/2 in BMDM in response to treatment with LPS. J: The effect of Oxy210 (5 μM) on the ratio of phosphorylated to total of ERK1/2 in BMDM in response to treatment with LPS. Densitometry of western blots (n = 4, biological replicates, ∗∗*P* < 0.001, *t*-test). K: Dose-dependence of the effect of Oxy210 on secretion of TNFα by BMDM in response to treatment with LPS for 4 h ∗∗∗*P* < 0.001 versus “no LPS”, *t*-test. L: Dose-dependence of the effect of Oxy210 on secretion of IL-6 by BMDM in response to treatment with LPS for 4 h ∗∗∗*P* < 0.001 versus “no LPS”, *t*-test. M:The effect of Oxy210 (5 μM) on expression of *il6* and *tnf* in BMDM in response to treatment with LPS for 2 h ∗*P* < 0.05, ∗∗*P* < 0.001 versus “no LPS”, *t*-test. Mean ± SEM are shown. LPS, lipopolysaccharide; BMDM, bone marrow-derived macrophage; FACS, fluorescence-activated cell sorting; TNFα, tumor necrosis factor α; IL-6, interleukin 6; ERK1/2, mitogen-activated protein kinase 1/2; NLRP3, NLR family pyrin domain containing 3; TLR, toll-like receptor.

Reduction in lipid raft abundance is generally associated with anti-inflammatory effects (6, 12, 54, 55). Therefore, we next examined the impact of Oxy210 on the functionality of the key inflammatory receptor TLR4. Murine BMDMs were treated with Oxy210 or vehicle for 18 h, followed by LPS stimulation for 15 min. Consistent with our earlier findings (Fig. 2E, F), flow cytometry analysis of CTxB staining confirmed that Oxy210 treatment reduced lipid raft abundance on the cell surface (Fig. 3A, B). TLR4 rapidly dimerizes and internalizes upon ligand activation and as expected, LPS stimulation led to a rapid loss of total cell surface TLR4 in control BMDMs (Fig. 3C, D). However, in Oxy210-treated BMDMs, LPS-induced TLR4 internalization was impaired (Fig. 3D). To further assess TLR4 activation, we used an antibody that specifically recognizes the nonactivated monomeric form of TLR4 (16). Following LPS stimulation, the proportion of monomeric TLR4 remaining on the surface of Oxy210-treated BMDM was significantly higher compared to LPS-treated control cells (Fig. 3E). Accordingly, when we quantified TLR4 dimerization, we found that approximately 75% of TLR4 was dimerized within 15 min of LPS stimulation in control BMDMs (Fig. 3F). In contrast, Oxy210 treatment impaired LPS-induced TLR4 dimerization, reducing it to approximately 20% (Fig. 3F).

Next, we examined the effect of Oxy210 on downstream TLR4-mediated signaling pathways. Treatment with Oxy210 (5 μM) reduced the abundance of NLR family pyrin domain containing 3 (NLRP3) (Fig. 3G, H). A similar reduction was observed for phosphorylated ERK1/2 (pERK1/2), while total ERK1/2 levels remained unchanged (Fig. 3I, J). In addition, Oxy210 suppressed LPS-induced secretion of TNFα (Fig. 3K) and IL-6 (Fig. 3L) by BMDMs in a dose-dependent manner. Reduction of the secretion of both cytokines was accompanied with reduction in expression of the corresponding genes (Fig. 3M). These findings indicate that Oxy210 inhibits LPS-induced TLR4 dimerization, downstream signaling, and proinflammatory cytokine production in macrophages.

### Oxy210 and reverse cholesterol transport

**Fig. 4:**
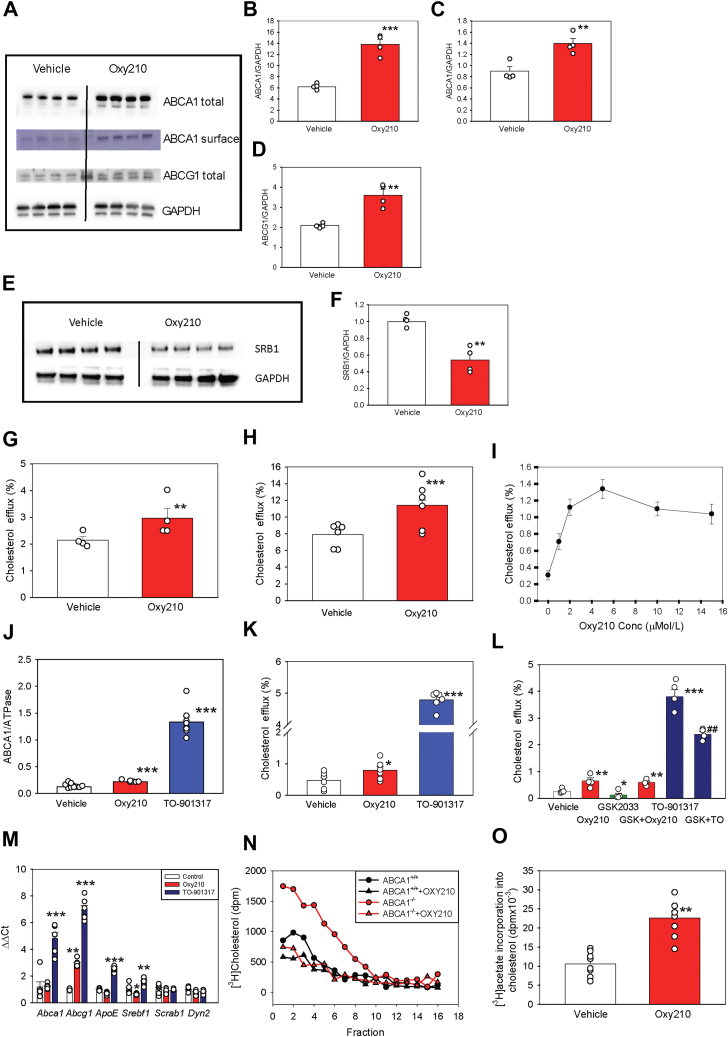
The effect of Oxy210 on reverse cholesterol transport. A: The effect of Oxy210 (5 μM) on the abundance of total and surface ABCA1 and ABCG1 in BMDM. Four biological replicates are shown in each Western blot. B: Quantitation (densitometry) of the abundance of total ABCA1 in BMDM as shown in (A)**.** C: Quantitation (densitometry) of the abundance of surface ABCA1 in BMDM as shown in A. D: Quantitation (densitometry) of the abundance of ABCG1 in BMDM as shown in (A). E: The effect of Oxy210 (5 μM) on the abundance of SRB1 in BMDM. Four biological replicates are shown in each western blot. F: Quantitation (densitometry) of the abundance of SRB1 in BMDM as shown in (E). G: The effect of Oxy210 (5 μM) on cholesterol efflux from BMDM to apoA-I. Cells were labeled with [^3^H] cholesterol, proportion of the label moved from cells to apoA-I (20 μg/ml) over subsequent 2 h incubation is shown. H: The effect of Oxy210 (5 μM) on cholesterol efflux from BMDM to HDL. Cells were labeled with [^3^H] cholesterol, proportion of the label moved from cells to HDL (30 μg/ml) over subsequent 2 h incubation is shown. I: Dose-dependence of the effect of Oxy210 on cholesterol efflux from RAW264.7 cells to apoA-I. Cells were labeled with [^3^H] cholesterol, proportion of the label moved from cells to apoA-I (20 μg/ml) over subsequent 2 h incubation is shown. J: The effect of Oxy210 (5 μM) and LXR agonist TO-901317 (4 μM) on the abundance of ABCA1 in RAW264.7 cells. Quantitated by densitometry of western blots. K: The effect of Oxy210 (5 μM) and LXR agonist TO-901317 (4 μM) on cholesterol efflux to apoA-I (20 μg/ml) from RAW 264.7 cells. Quantitated as described in (G). L: The effect of Oxy210 (5 μM), LXR antagonist GSK2033 (1 μM), and LXR agonist TO-901317 (4 μM) on cholesterol efflux to apoA-I (20 μg/ml) from RAW 264.7 cells. Quantitated as described in (G)**.** M: The effect of Oxy210 (5 μM) and LXR agonist TO-901317 (4 μM) on the expression of LXR-dependent and LXR-independent genes in BMDM (RT -PCR). N: The effect of Oxy210 (5 μM) on lipid raft abundance in ABCA^+/+^ and ABCA1^−/−^ fibroblasts. Plasma membranes isolated from cells labeled with [^3^H] cholesterol were fractionated by density gradient centrifugation as described in “Methods”. Lipid rafts were defined as fractions with highest [^3^H] cholesterol and flotillin-1 content. *P* < 0.05 (Mann-Whitney test); area under the curve values are shown in supplemental Table S2. O: The effect of Oxy210 (5 μM) on the rate of cholesterol biosynthesis in BMDM ([^3^H] acetate incorporation in cholesterol over 4 h). All graphs: Mean ± SEM are shown. ∗*P* < 0.05, ∗∗*P* < 0.01; ∗∗∗*P* < 0.001 versus vehicle (*t*-test). ^##^*P* < 0.01 versus TO-901317 (ANOVA). BMDM, bone marrow-derived macrophage; LXR, liver X receptor; apoA-I, apolipoprotein A-I; ABCG1, ATP binding cassette transporter G1; ABCA1, ATP binding cassette transporter A1.

Several oxysterols were identified as agonists of the liver X receptors (LXRs), key modulators of the expression of ABCA1 and ABCG1, cholesterol transporters responsible for cholesterol efflux (24, 56). LXR activation would be a plausible mechanism explaining the effects of Oxy210 on lipid rafts. Indeed, treatment of BMDM with Oxy210 doubled the abundance of total ABCA1 (Fig. 4 A, B) and increased the abundance of cell-surface ABCA1 by 50% (Fig. 4A, C); it also doubled the abundance of ABCG1 (Fig. 4A, D). In contrast, the abundance of scavenger receptor B1 (SR-B1), cholesterol transporter not regulated by LXR, was reduced by 50% (Fig. 4E, F). Consistent with elevation of ABC transporters, cholesterol efflux from BMDM to purified apolipoprotein A-I (apoA-I) (Fig. 4G) or isolated HDL (Fig. 4H), were increased by about 1.5-fold.

In contrast to BMDMs, RAW264.7 murine macrophages have very low basal level of ABCA1, providing an advantage for testing a condition expected to increase cholesterol efflux. Dose-dependence of the effect of Oxy210 on cholesterol efflux is shown in Fig. 4I, maximum effect of Oxy210 on cholesterol efflux was achieved at 5 μMol/L. While the total ABCA1 abundance in RAW 264.7 cells almost doubled after treatment with Oxy210, treatment of the same cells with a synthetic LXR agonist TO-901317 (4 μM) was more effective, elevating ABCA1 abundance by 10-fold (Fig. 4J). Cholesterol efflux from Oxy210-treated RAW264.7 cells to apoA-I increased 1.5-fold while treatment with TO-901317 caused a 10-fold elevation of cholesterol efflux (Fig. 4K) showing that the effects of Oxy210 on reverse cholesterol transport were much weaker than a classical LXR agonist. Furthermore, LXR antagonist, GSK2033 (57), failed to reduce elevation of cholesterol efflux caused by Oxy210, while reducing elevation of cholesterol efflux by LXR agonist TO-901317 (Fig. 4L). Finally, RT-PCR was used to directly assess the effects of Oxy210 and TO-901317 on the expression of four LXR-activated genes - *Abca1, Abcg1, ApoE*, and *Srebf1* (58) - in BMDMs, along with two LXR-independent control genes, *Scrab1* and *Dyn2*. While TO-901317 strongly upregulated all four LXR-dependent genes without affecting the LXR-independent controls, Oxy210 only modestly increased *Abcg1* expression (Fig. 4M). These results suggest that the Oxy210-induced increase in ABCA1 abundance and cholesterol efflux occurs independently of LXR activation.

To examine the role of ABCA1 in Oxy210-induced lipid raft reduction, we compared its effects in fibroblasts from a patient with Tangier disease, a genetic disorder characterized by lack of functional ABCA1, with those from a healthy individual. Previously, we confirmed the absence of ABCA1 and the lack of cholesterol efflux to apoA-I in Tangier fibroblasts (59). *ABCA1*^*−/−*^ fibroblasts exhibited a higher abundance of lipid rafts than *ABCA1*^*+/+*^ cells (Fig. 4N). However, Oxy210 effectively reduced lipid rafts abundance in both *ABCA1*^*+/+*^ and *ABCA1*^*−/−*^ cells (Fig. 4L and supplemental Table S2). These findings indicate that ABCA1 is not required for Oxy210-mediated lipid raft reduction.

Another potential mechanism we considered was that the reduction of lipid rafts was a consequence of inhibition of cholesterol biosynthesis by Oxy210. Statins, competitive inhibitors of cholesterol biosynthesis (60), as well as genetic defects of cholesterol biosynthesis (61) reduce the abundance of lipid rafts. However, Oxy210 doubled the rate of cholesterol biosynthesis in BMDMs, effectively ruling out this possibility (Fig. 4O).

### Oxy210 and cytoskeleton

In several previous studies, we demonstrated that changes in the abundance of lipid rafts may be independent from cholesterol efflux (47, 53, 62). Instead, the driving force in lipid raft regulation was cytoskeletal changes mediated by small GTPase Cdc42.

Abundance of Cdc42 was not affected by Oxy210 (supplemental Fig. S3A). Transfection of RAW264.7 cells with siRNA^Cdc42^ resulted in about 80% reduction of Cdc42 abundance (supplemental Fig. S3A). Although cholesterol efflux from RAW264.7 cells to apoA-I was sharply reduced by Cdc42 silencing (supplemental Fig. S3B), Oxy210 was able to stimulate cholesterol efflux from both control and Cdc42-silenced cells to a similar degree. Silencing of Cdc42 increased the abundance of ABCA1 in the cells; however, Oxy210 increased ABCA1 abundance independent of Cdc42 silencing (supplemental Fig. S3C). Finally, silencing of Cdc42 had no statistically significant effect on either the abundance of lipid rafts or the ability of Oxy210 to reduce it (supplemental Fig. S3D and supplemental Table S4). We conclude that the effects of Oxy210 are independent of Cdc42.

### Oxy210 and lipid raft composition

**Fig. 5:**
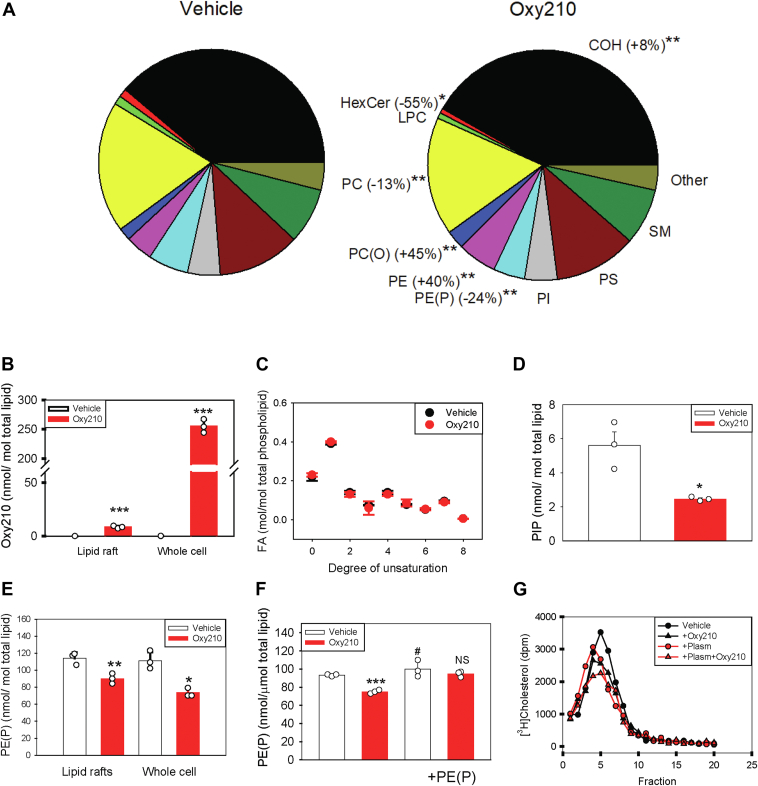
The effect of Oxy210 on lipid raft lipidome. A–F: Lipids extracted from BMDM whole cell lysate or isolated lipid rafts were analyzed using liquid chromatography/mass-spectrometry (lipidomics) as described in “Methods”. All concentrations were normalized to the level of PC. A**:** The effect of Oxy210 (5 μM) on relative abundance of major lipid classes in lipid rafts. Numbers show differences in the abundance of individual lipid classes in lipid rafts isolated from Oxy210-treated versus vehicle-treated cells.B: Abundance of Oxy210 in lipid rafts and whole cell lysate after treatment with Oxy210 (5 μM). C: The effect of Oxy210 (5 μM) on degree of saturation (number of double bonds) of fatty acids chains in raft lipids. D: The effect of Oxy210 (5 μM) on the abundance of PIP in lipid rafts. E: The effect of Oxy210 (5 μM) on PE(P) levels in the lipid rafts and whole cell. F: The effect of Oxy210 (5 μM) on PE(P) levels in whole cells before and after loading of cells with PE(P) (20 μM, 18 h). G: The effect of Oxy210 (5 μM) and PE(P) loading (20 μM, 18 h) on lipid raft abundance. Plasma membranes isolated from cells labeled with [^3^H] cholesterol were fractionated by density gradient centrifugation as described in “Methods”. Lipid rafts were defined as fractions with highest [^3^H] cholesterol and flotillin-1 content. Area under the curve values and statistics are shown in supplemental Table S4. Mean ± SEM are shown. ∗*P* < 0.05, ∗∗*P* < 0.01, ∗∗∗*P* < 0.001 (versus vehicle); #*P* < 0.05 (versus vehicle w/o PE(P) loading, *t*-test).COH, cholesterol; HexCer, monohexosylceramide; LPC, lysophosphatidylcholine; PC, phosphatidylcholine; PC(O), alkyl phosphatidylcholine; PE, phosphatidylethanolamine; PE(O), alkyl phosphatidylethanolamine; PE(P), alkenyl phosphatidylethanolamine (plasmalogen); PIP, phosphatidylinositol monophosphate; PI, phosphatidylinositol; PS, phosphatidylserine; SM, sphingomyelin; BMDM, bone marrow-derived macrophage.

Lipid rafts are dynamic structures, and their lipid composition is a major determinant of stability and, consequently, abundance. Therefore, we assessed the effect of Oxy210 on the lipidome of lipid rafts isolated from BMDMs. Figure 5A shows the abundance of the major lipid classes in lipid rafts in cells treated with Oxy210 or vehicle. The abundances of individual lipids were normalized to the total lipid content of the fraction, thus showing relative changes in the lipid composition of the fraction. Percentage change (shown in brackets) refers to the difference between relative abundance of a lipid in Oxy210-treated versus untreated cells.

First, we investigated whether Oxy210 integrates into lipid rafts and alters their stability by displacing other lipids or disrupting raft structure, similar to 7-ketocholesterol (63) or curcumin (64). However, our analysis revealed that the relative abundance of Oxy210 in the cell lysate was 30-fold higher than in lipid rafts (Fig. 5B). This suggests that Oxy210 is largely excluded from lipid rafts, making it unlikely that its effects on raft stability results from direct incorporation into their structure.

The stability of lipid rafts is primarily determined by the abundance of cholesterol and sphingomyelin, as well as the degree of saturation of phospholipid fatty acid chains (3). Treatment with Oxy210 did not alter the relative abundance of sphingomyelin (Fig. 5A) or the degree of fatty acid saturation (Fig. 5C). However, Oxy210 increased cholesterol abundance by 8% (Fig. 5A), which would be expected to stabilize lipid rafts. Notably, treatment with Oxy210 did not significantly affect cellular cholesterol content (35.2 ± 0.1% versus 36.6 ± 0.3% for Oxy210 and vehicle treatment, respectively (Mean ± SEM), *P* = 0.08, n = 3).

We then investigated if the abundance of any of the lipid species previously implicated in changes of lipid raft stability or function was significantly affected by Oxy210. Two such lipids were identified: plasmalogen (PE(P)) (65) (Fig. 5A) and PIP (62) (Fig. 5D).

The concentration of PE(P), the predominant plasmalogen in the rafts, was similar in the rafts and in the whole cell lysate and the effect of Oxy210 on PE(P) levels in rafts and whole cells was also similar (Fig. 5E), indicating that level of PE(P) in lipid rafts reflects cellular level of this lipid. To reverse the effect of Oxy210 on the abundance of PE(P), we loaded cells with exogenous PE(P). The loading resulted in a modest increase of cellular PE(P) level, however, this was sufficient to reverse the effect of Oxy210 on this lipid (Fig. 5F). Loading cells with PE(P) had no effect on the abundance of lipid rafts, nor did it affect the influence of Oxy210 on lipid rafts (Fig. 5G and supplemental Table S4). These findings make it unlikely that the effect of Oxy210 on PE(P) level in lipid rafts is a major contributor to its ability to reduce the abundance of the rafts. This leaves PIP as the only raft lipid that may be responsible for the changes of their stability, this will be discussed below.

### Oxy210 and recycling of lipid rafts

Yet another pathway regulating lipid raft abundance on the cell surface is their endocytosis and recycling (66). The key element regulating endocytosis and recycling of lipid rafts is the small GTPase Arf6 (66, 67). We have previously documented that Arf6 is also involved in recycling of ABCA1 (68). Silencing of Arf6 impaired the ABCA1 endocytosis preserving ABCA1 on the cell surface and stimulating cholesterol efflux. Reduction of Arf6 also impaired the recycling of lipid rafts reducing their abundance on the cell surface (67). These observations led us to hypothesize that an effect on recycling and endocytosis might be involved in the action of Oxy210.

**Fig. 6:**
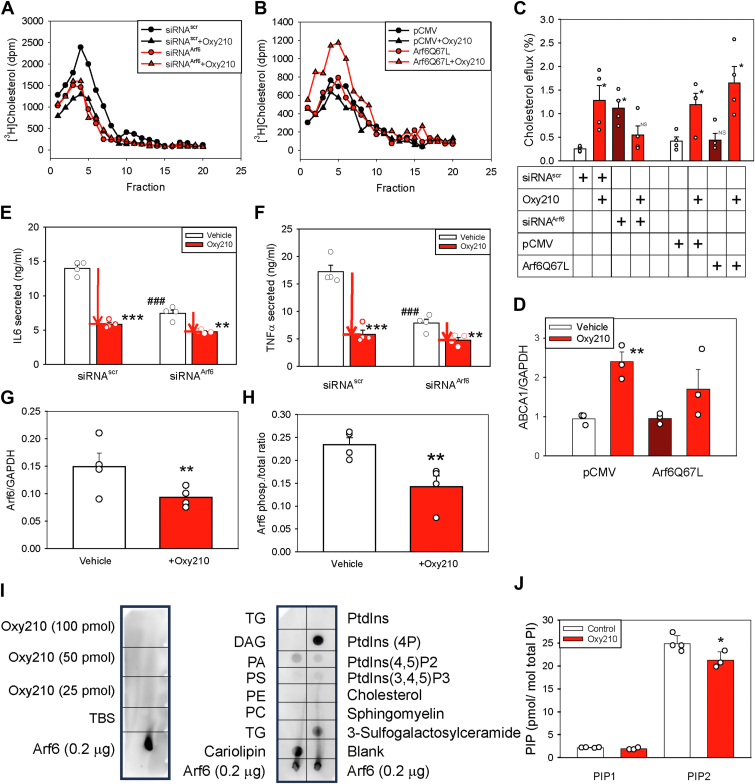
Mechanism of the effects of Oxy210 in RAW264.7 cells. A: The effect of Arf6 silencing and of Oxy210 (5 μM) on lipid raft abundance. Plasma membranes isolated from cells labeled with [^3^H] cholesterol were fractionated by density gradient centrifugation as described in “Methods”. Lipid rafts were defined as fractions with highest [^3^H] cholesterol and flotillin-1 content; *P* < 0.05 (Mann-Whitney test), Area under the curve values are shown in supplemental Table S5. B: The effect of Arf6Q67L overexpression and of Oxy210 (5 μM) on lipid raft abundance. Methodology is the same as in A, *P* < 0.001 (Mann-Whitney test). Area under the curve values are shown in supplemental Table S6. C: The effect of Arf6 silencing and overexpression and Oxy210 (5 μM) on cholesterol efflux to apoA-I. Cells were labeled with [^3^H] cholesterol, proportion of the label moved from cells to apoA-I (20 μg/ml) over subsequent 2 h incubation is shown. Mean ± SEM; ∗*P* < 0.05; versus siRNA^scr^ or pCMV; (ANOVA, n = 4). D: The effect of Arf6 overexpression and Oxy210 (5 μM) on the ABCA1 abundance. The ABCA1 abundance was quantified by densitometry of western blots (shown in supplemental Fig. S5B) and was normalized to the abundance of ABCA1 in untransfected cells. Mean ± SEM; ∗∗*P* < 0.01; versus pCMV; (ANOVA, n = 3). E: The effect of Arf6 silencing and Oxy210 (5 μM) on LPS-induced secretion of IL6. After Arf6 silencing, cells were stimulated with LPS (100 ng/ml) for 20 h in the presence or absence of Oxy210; medium was collected and subjected to analysis. Arrows depict the level of inhibition by Oxy210. Mean ± SEM;∗∗*P* < 0.01 versus vehicle, ∗∗∗*P* < 0.001 versus vehicle, ^###^*P* < 0.001 versus siRNA^scr^; (n = 4, *t*-test). F: The effect of Arf6 silencing and Oxy210 (5 μM) on LPS-induced secretion of TNFα. After Arf6 silencing, cells were stimulated with LPS (100 ng/ml) for 20 h in the presence or absence of Oxy210; medium was collected and subjected to analysis. Arrows depict the level of inhibition by Oxy210. Mean ± SEM;∗∗*P* < 0.01 versus vehicle, ∗∗∗*P* < 0.001 versus vehicle, ^###^*P* < 0.001 versus siRNA^scr^; (n = 4, *t*-test). G: The effect of Oxy210 (5 μM) on the abundance of Arf6. Quantitated by densitometry of western blots shown in supplemental Fig. S4C. Mean ± SEM; ∗∗*P* < 0.01; versus vehicle (n = 4, *t*-test). H: The effect of Oxy210 (5 μM) on the ratio of phosphorylated to total Arf6. Mean ± SEM; ∗∗*P* < 0.01; versus vehicle (n = 4, *t*-test). Quantitated by densitometry of western blots shown in supplemental Fig. S4D. I: Binding of Arf6 to Oxy210 in protein lipid overlay assay. Indicated amounts of Oxy210, Arf6 (0.2 μg) or vehicle (TBS) were applied (2 μl/dot) on a nitrocellulose membrane (left) or a commercial membrane with preloaded lipids was used (right). Both membranes were incubated for 1 h in the blocking solution and then overnight at 4^°^C with recombinant human His-Arf6 (1 μg/ml), followed by 2 h incubation with anti-His Tag monoclonal antibody (1:1000). J: The effect of Oxy210 (5 μM) on the abundance of PIP1 and PIP2. Lipids extracted from RAW264.7 cell whole cell lysate or isolated lipid rafts were analyzed using liquid chromatography/mass-spectrometry (lipidomics) as described in “Methods”. All concentrations were normalized to the level of PC. Mean ± SEM; ∗*P* < 0.05; versus vehicle (n = 4, *t*-test). LPS, lipopolysaccharide; TNFα, tumor necrosis factor α; apoA-I, apolipoprotein A-I; ABCA1, ATP binding cassette transporter A1; Arf6, ARF GTPase 6.

Silencing of Arf6 with siRNA reduced Arf6 abundance (Fig. S4A) and reduced abundance of lipid rafts by 40% (Fig. 6A). Treatment of cells with Oxy210 reduced the abundance of lipid rafts, but the same treatment combined with Arf6 silencing resulted in a loss of the effect of Oxy210 (Fig. 6A and supplemental Table S5). We then tested the effect of overexpression of Arf6. To avoid a possible confounding effect of changes in Arf6 activity, we used a constitutively active (GTP hydrolysis-resistant) variant, Arf6Q67L (69, 70). Overexpression of Arf6Q67L did not affect the abundance of lipid rafts, but treatment of cells overexpressing Arf6Q67L with Oxy210 not only failed to reduce the abundance of the rafts, but unexpectedly elevated it (Fig. 6B and supplemental Table S6).

Consistent with our previous findings (68), Arf6 silencing stimulated cholesterol efflux to a similar extent as Oxy210; however, Oxy210 failed to stimulate cholesterol efflux from cells with silenced Arf6 (Fig. 6C). Overexpression of constitutively active variant, Arf6Q67L led to an increase in the abundance of total Arf6 in cells (supplemental Fig. S4B), but did not affect ABCA1 abundance or cholesterol efflux (Fig. 6C, D and supplemental Fig. S4B). Oxy210 increased ABCA1 abundance and cholesterol efflux from cells without Arf6 overexpression and, unexpectedly, in cells overexpressing Arf6Q67L (Fig. 6C, D and supplemental Fig. S4B).

Silencing of Arf6 reduced LPS-stimulated secretion of IL6 and TNFα from macrophages by about 50% (Fig. 6E, F). Treatment of cells with Oxy210 further reduced secretion of both cytokines; however, the effect of Oxy210 on cells with Arf6 silencing was half of that on cells without Arf6 silencing (Fig. 6E, F red arrows). Thus, Arf6 silencing partially blocked the effect of Oxy210 on the marker of inflammation. An incomplete effect is likely due to the presence of residual Arf6; however, a possibility of the contribution of another mechanism cannot be ruled out.

We next tested the effect of Oxy210 on the abundance of total and activated (phosphorylated) Arf6. Analysis of the abundance of total Arf6 in cell lysates by western blot showed that treatment with Oxy210 reduced total abundance of Arf6 by 36% (Fig. 6G and supplemental Fig. S4C). Pull-down assay separating phosphorylated (activated) Arf6 from total Arf6, showed that the proportion of activated Arf6 (ie ratio of abundancies of phosphorylated to total Arf6) was also reduced by 40% after treatment with Oxy210 (Fig. 6H and supplemental Fig. S4D).

To investigate the possibility of a direct interaction between Oxy210 with Arf6, we employed a protein–lipid overlay assay that detects the binding of purified proteins to individual lipids immobilized on a membrane (50). We previously used this assay to detect the binding of apoA-I binding protein (AIBP) to PIP1 (62). Although Arf6 showed strong binding to PIP1, cardiolipin, and 3-sulfogalactosiylceramide, no binding of Arf6 to Oxy210 was detected (Fig. 6I). Recognizing the limitations of this assay, such as its inability to account for the potential need for cofactors or the exposure of the binding site on the immobilized partner, this finding does not support a direct interaction between Oxy210 and Arf6.

Arf6 is involved in several cellular regulatory networks that may connect it to lipid rafts, including the ability of Arf6 to activate PIP kinase (PIPK), which converts PIP1 into PIP2 (71), the latter being a recognized downstream effector of Arf6 (72, 73). Original lipidomics analysis indicated that total PIP in lipid rafts was reduced in Oxy210-treated cells (Fig. 5D) prompting us to analyze the effect of Oxy210 on the abundance of PIPs in BMDMs using a lipidomics analysis capable of distinguishing between phosphorylated isoforms of PIPs. We found that while the abundance of PIP1 was unaffected, the abundance of PIP2 was significantly reduced by Oxy210 (15% reduction) (Fig. 6J). It must be noted that mass spectrometry cannot distinguish between three isoforms of PIP2,—PI(3,4)P_2_, PI(3,5)P and PI(4,5)P_2_,—but of those only PI(4,5)P_2_ is known to be involved in regulation of lipid rafts. These findings point to the lipid raft recycling as a possible mechanism responsible for the modification of lipid rafts by Oxy210.

## Discussion

Lipid raft therapy is a rapidly developing therapeutic approach (54). The rationale for this strategy is based on the key role of lipid rafts in regulating various pathways across a diverse range of diseases, including neurodegeneration (11), infectious diseases (9), cancer (74) and cardiovascular diseases (12) to name a few. Most pathological conditions are associated with an overabundance of lipid rafts, while lipid raft deficiency is rare. This led to the introduction of the concept of “inflammaraft”, which describes a subset of “pathological” lipid rafts with increased abundance and/or stability and elevated inflammatory receptor content (15, 55). It has been hypothesized that these pathological lipid rafts appear in diseased states and can be discerned and targeted selectively from “physiological” rafts. Accordingly, therapeutic approaches aimed at a graded lowering of the abundance of pathological lipid rafts while leaving physiological lipid rafts intact have been successful in experimental settings (for review, see (54)). While this supports the inflammaraft concept, the mechanisms of inflammaraft formation are not well defined and seem to be diverse (55). In this study, we mechanistically investigated a novel compound, a synthetic oxysterol, which proved to be an efficient lipid raft modifying tool but was chemically different from previously tested forms of lipid raft-targeting agents.

In this study, we demonstrated that Oxy210 effectively reduced the abundance of lipid rafts in various cells: murine and human, cell lines and primary cells, blood cells and neurones. Importantly, Oxy210 was able to gradually reduce the abundance of lipid rafts and in the experiments where abundance of lipid rafts was elevated, *eg* by treatment with LPS or following deletion of ABCA1, addition of Oxy210 mitigated elevation of lipid rafts reducing their abundance to the level similar to that in control cells. Inflammatory markers elevated in response to LPS, such as abundance and dimerization of TLR4 or abundance of NLRP3, were reduced by Oxy210 to the level slightly above those in controls. Given important physiological role of lipid rafts, this property reduces the probability of adverse effects and indeed, no adverse effects were noted in either in vitro or in vivo experiments described in this report or in previous publications (31, 75). Gradual reduction of lipid rafts is similar to the effect of another promising form of lipid raft therapy, AIBP, and is consistent with the concept of inflammaraft—a “pathological” raft with different properties and different susceptibility to intervention (15).

Reduction of lipid rafts was accompanied by mitigation of inflammation. In vitro*,* Oxy210 reduced the abundance of cell surface TLR4 as well as its dimerization and internalization in response to LPS. Accordingly, downstream effects of TLR4 activation, abundance of NLRP3 and secretion of cytokines, were also reduced in LPS-treated macrophages. In vivo*,* Oxy210 reduced the abundance of neutrophils and Ly6C^lo^ (patrolling) monocytes in blood without affecting the proportion of Ly6C^hi^ (inflammatory) monocytes. Oxy210 had no effect on the parental GMP population in the bone marrow, suggesting the changes do not reflect a defect in myeloid development. We speculate that since Ly6^lo^ monocytes arise from Ly6^hi^ in circulation (76), the selective effect might reflect slowing of monocyte maturation. When inflammation was induced in a model of acute peritonitis, Oxy210 mitigated elevation of the abundance of TLR4 on the surface of neutrophils and Ly6C^hi^ monocytes, as well as secretion of TNFα and IL-6 from in vitro activated peritoneal macrophages. TNFα and IL-6 levels in plasma of mice treated with LPS were also reduced by Oxy210. There was no effect of Oxy210 on hematopoietic precursors in the bone marrow, as larger doses of LPS and longer time points are needed to trigger emergency myelopoiesis. Thus, in vitro*,* as well as in an in vivo model of acute peritonitis, Oxy210 effectively mitigated inflammation at the level of blood cells and peritoneum.

Several mechanisms of lipid raft disruption were previously described (for review see (54)). One is based on the depletion of lipid species that determine lipid raft stability: cholesterol, sphingomyelin, and saturated fatty acids. Consequently, depletion of cholesterol using synthetic nonspecific cholesterol acceptors, such as methyl-β-cyclodextrin (MβCD), stimulation of cholesterol efflux by LXR agonists, and reduction of cholesterol biosynthesis by statins was effective in reducing raft cholesterol content and consequently their stability and abundance. However, in cells treated with Oxy210, cholesterol content of lipid rafts was elevated, while sphingomyelin content and the proportion of unsaturated fatty acid were unaffected. There was no activation of *Abca1* transcription and while ABCA1 abundance and cholesterol efflux were elevated by treatment with Oxy210, it did not require ABCA1 or cholesterol efflux for its activity to reduce lipid rafts. In addition, Oxy210 did not inhibit cholesterol biosynthesis, but instead enhanced it, likely in response to enhanced cholesterol efflux. Another possible mechanism was modulation of lipid raft abundance through changes to the cytoskeleton (47, 53, 62). However, the action of Oxy210 did not depend on the abundance of Cdc42, a key element connecting lipid rafts with pathways regulating β-actin polymerization. Incorporation of Oxy210 into lipid rafts was minimal, in effect, it was selectively excluded from lipid rafts, making it unlikely that Oxy210 changes stability of lipid rafts by a membrane-active mechanism. Other interesting change in lipid raft composition in response to Oxy210 treatment was a reduction of the relative abundance of plasmalogen, which stabilizes lipid rafts (65). However, loading cells with exogenous plasminogen to restore its levels, did not prevent reduction in lipid raft abundance by Oxy210, ruling out this mechanism of reducing lipid rafts.

**Fig. 7:**
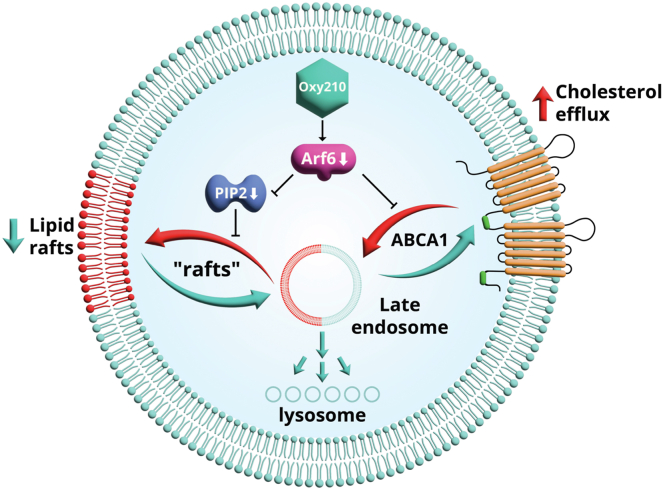
Schematic representation of the proposed mechanism of action of Oxy210. Plasma membrane components are constantly recycling between the membrane and intracellular compartments. During recycling they pass through a checkpoint in late endosomes, where components of “disordered” regions of plasma membrane are directed to lysosomes, while “ordered” ensembles are directed to the plasma membrane lipid rafts. Small GTPases, including Arf6, are proposed to play key role in this regulatory checkpoint through activation of phosphatidylinositol 4-phosphate 5-kinase (PIP5K), which catalyzes conversion of PIP1 to PIP2. Arf6 is also involved in sorting of internalized ABCA1; however, it works in an opposite direction trafficking ABCA1 for degradation in lysosomes. We propose that Oxy210 reduces the abundance and activity of Arf6, which in turn decreases the activity of PIP5K, the abundance of PIP2 and the recycling of lipid rafts to the plasma membrane. Suppression of Arf6 by Oxy210 also inhibited internalization and degradation of ABCA1, increasing its abundance on the cell surface and consequently increasing the rate of cholesterol efflux. ABCA1, ATP binding cassette transporter A1; Arf6, ARF GTPase 6.

Another, much underappreciated mechanism of lipid raft regulation is their cycling between recycling endosomes and the cell surface, a process regulated by small GTPase Arf6 (66, 67). The hypothesis, originally proposed by Balasubramanian *et al.* (67) and supported by a number of studies (73, 77), stipulates that during recycling, both recycled and newly synthesized components of the plasma membrane have to pass through a checkpoint in late endosomes. At this checkpoint, components of “disordered” regions of plasma membrane are directed to lysosomes, while “ordered” ensembles are directed to the plasma membrane lipid rafts. Small GTPases, including Arf6, are proposed to play key role in this regulatory checkpoint. The mechanism of regulation of endocytosis and exocytosis likely involves activation by Arf6 of phosphatidylinositol 4-phosphate 5-kinase (PIP5K), which catalyzes conversion of PI(4)P_1_ to PI(4,5)P_2_, a recognized regulator of endocytosis and exocytosis (72). We recently demonstrated that Arf6 is involved in sorting of internalized ABCA1 (68). However, it works in an opposite direction trafficking ABCA1 for degradation in lysosomes (68). The results of this study are consistent with both hypotheses. We propose that Oxy210 reduces the abundance and activity of Arf6, which in turn decreases the activity of PIP5K, the abundance of PI(4,5)P and the recycling of lipid rafts to the plasma membrane (Fig. 7). Suppression of Arf6 by Oxy210 also inhibited internalization and degradation of ABCA1, increasing its abundance on the cell surface and consequently increasing the rate of cholesterol efflux.

Endocytic recycling is a relatively slow process, which may seem incompatible with the view of lipid rafts as transient structures with sub-second lifetimes. However, this short lifetime is characteristic of lipid rafts observed in model membranes. In contrast, cellular lipid rafts are stabilized by resident proteins, membrane curvature, and raft aggregation, resulting in more stable structures (78). These stabilized rafts exhibit lifetimes compatible with slower processes such as endocytosis, pathogen internalization, and aggregation of amyloidogenic proteins (78, 79). Moreover, once a trafficking vesicle is formed, its lipid and protein composition remain stable during transit and is unaffected by the composition of other membranes it may encounter. Therefore, the duration of intracellular transport is not a limiting factor for the proposed model (77).

Our findings are consistent with the reports documenting a key role of Arf6 in pathogenesis of various inflammatory conditions, such as sepsis (80), arthritis (80), neuroinflammation (81), asthma (82) and atherosclerosis (83), inhibition of Arf6 was anti-inflammatory in these conditions. Many of the pathways responsible for these disorders are likely to include elements that are lipid raft-dependent (80, 82, 84, 85), consistent with potentially broad applicability of therapeutic targeting of the proposed mechanism.

### Limitations of the study

Although our findings support the key elements of the Arf6-centered recycling-focused mechanisms of Oxy210-mediated activity on lipid rafts, there are several limitations. Most importantly, the mechanism by which Oxy210 inhibits Arf6 abundance and activation remains unidentified, making it difficult to explain the unexpected finding that Oxy210 elevated raft abundance in cells overexpressing the constitutively active mutant of Arf6. Furthermore, it is unclear whether the opposite effects of Arf6 inhibition on lipid rafts and ABCA1 are mediated by similar or coordinated mechanisms, and what contribution each mechanism makes to the anti-inflammatory effects of Oxy210. Additionally, since Arf6 is involved in a broad range of pathways, not all of which are lipid raft-dependent (86), it is challenging to rule out the potential contribution of lipid raft-independent pathways to the anti-inflammatory activity of Oxy210. It is important to recognize that both Arf6 and lipid rafts are essential elements of physiological regulatory networks and defining the exact conditions to achieve the intended therapeutic effects while not compromising normal physiology may potentially be challenging, a limitation Oxy210 shares with other forms of lipid raft therapy.

In conclusion, we identified a novel mechanism regulating lipid raft abundance, in which the synthetic oxysterol Oxy210 targets the small GTPase Arf6 and modulates the recycling of lipid rafts between the cell surface and endosomes. This process inhibits lipid raft abundance and mitigates the inflammatory response.

## Data Availability

Oxy210 is available from Farhad Parhami (fparhami@maxbiopharma.com). All other unique/stable reagents generated in this study are available from the lead contact, Dmitri Sviridov (Dmitri.Sviridov@Baker.edu.au), with a completed materials transfer agreement. Flow cytometry, lipidomics, microscopy, and western blot data are available upon request. Any additional information required to reanalyze the data reported in this paper is available from the lead contact upon request.

## Supplemental data

This article contains supplemental data: supplemental Figs. S1–S5 and supplemental Tables S1–S6.

## Conflict of interest

Farhad Parhami is an employee of and holds shares in MAX BioPharma, Inc, a company with a commercial interest in drug discovery and development. All the other authors declare that they have no conflicts of interest with the contents of this article.
