# Supplementary material for: Targeting the ARF6-dependent recycling pathway to alter lipid rafts and reduce inflammation
Source: J Lipid Res. 2025 Sep 16;66(10):100900. doi: 10.1016/j.jlr.2025.100900 (PMC12552991; doi:10.1016/j.jlr.2025.100900)
Supplement: Supporting information [file mmc1.pdf]

Targeting the ARF6-dependent recycling pathway to alter lipid rafts and reduce inflammation

Nigora Mukhamedova, Andrew J. Fleetwood, Kevin Huynh, Yangsong Xu, Tilly Van Buuren-Milne, Alexandra Faulkner, Ying Fu, Farhad Parham[2], Peter J. Meikle, Ilya Levental, Michael Bukrinsky, Andrew J. Murphy, Dmitri Sviridov.

Supporting Information

(Figures S1-S5 and tables S1-S6)

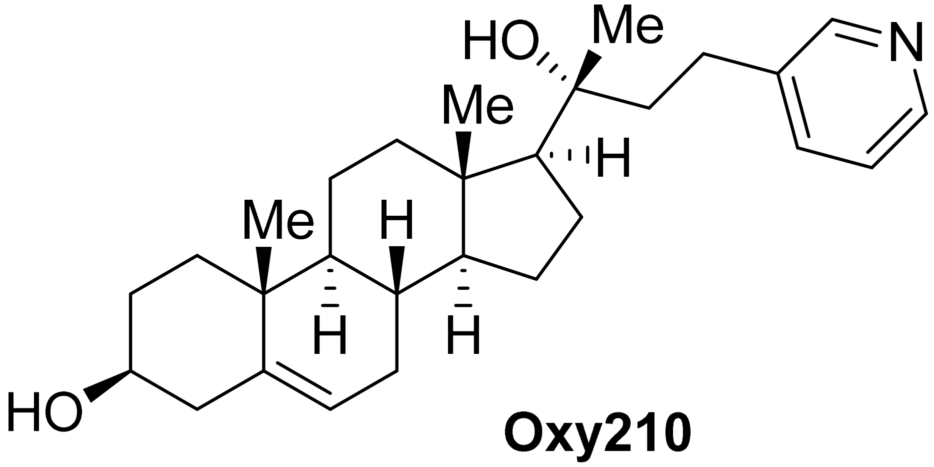

Oxy210

Chemical Formula: $C_{28}H_{41}NO_2$
Molecular Weight: 423.64

Oxy210: (*3S,8S,9S,10R,13S,14S,17S*)-17-((*R*)-2-hydroxy-4-(pyridin-3-yl)butan-2-yl)-10,13-dimethyl-2,3,4,7,8,9,10,11,12,13,14,15,16,17-tetradecahydro-1H-cyclopenta[a]phenanthren-3-ol

Figure S1. Structure of Oxy210

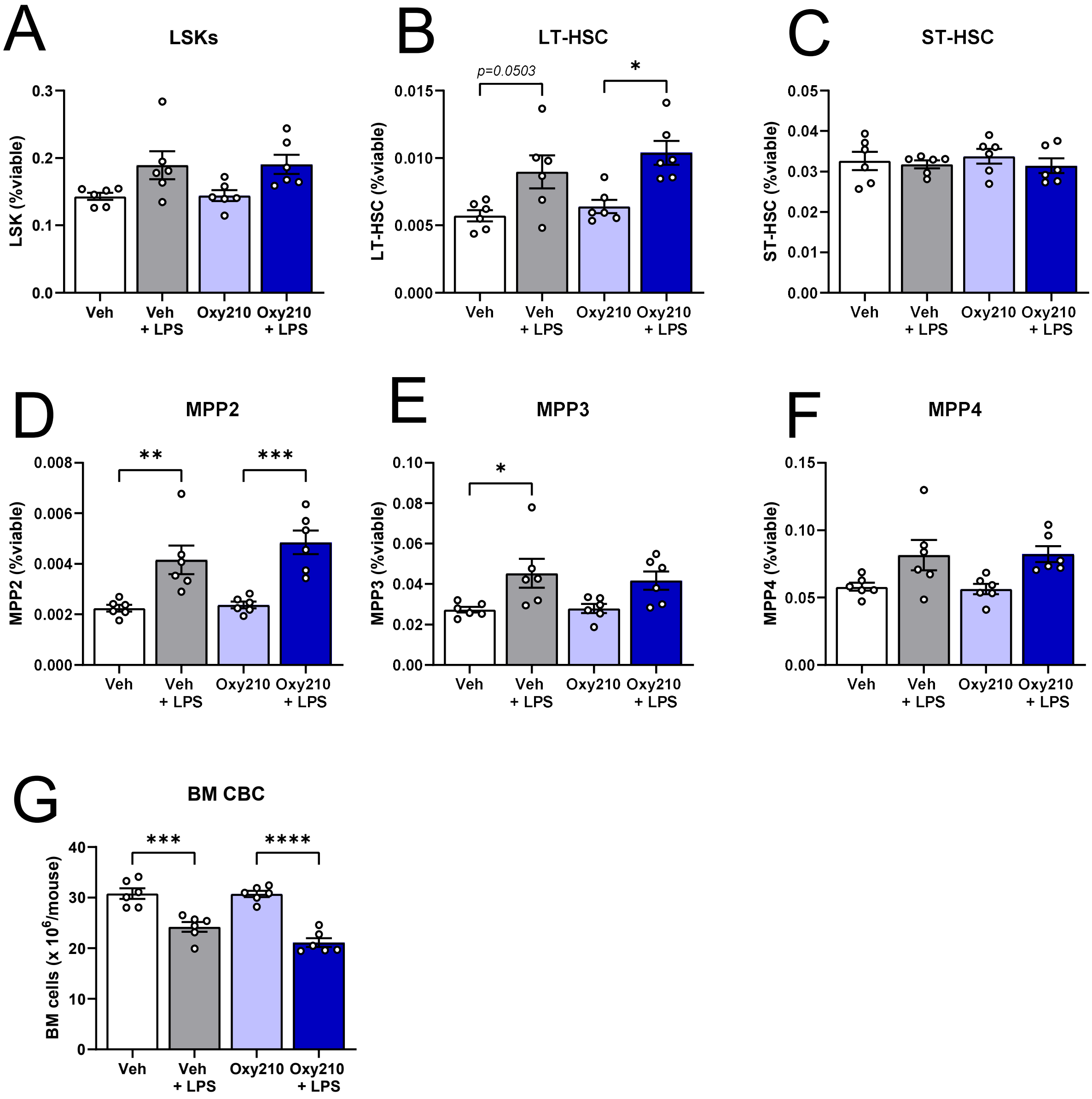

Figure S2. The effect of Oxy210 on hematopoietic stem and progenitor cells

Wild-type mice were fed Oxy210 or vehicle (Veh) control for 4 days. On day 4, a subset of animals was challenged with LPS (5 µg/mouse; i.p.), and an analysis of BM hematopoietic stem and progenitor cells was performed 2 hours later.

A-F. Cell frequency (% of viable cells) of **(A)** LSKs ($Lin^{-}Sca1^{+}cKit^{+}$), **(B)** LT-HSC ($CD48^{-}CD150^{+}LSK$), **(C)** ST-HSC ($CD48^{-}CD150^{-}LSK$), **(D)** MPP2 ($Flt3^{-}CD48^{+}CD150^{+}LSK$), **(E)** MPP3 ($Flt3^{-}CD48^{+}CD150^{-}LSK$), and **(F)** MPP4 ($Flt3^{+}CD48^{+}CD150^{-}LSK$) in the BM of mice (n=6 mice/group).

G. Total white blood cell counts in bone marrow of mice as treated above.

Data are presented as mean ± SEM. Data points represent individual mice. Significance was determined by two-way ANOVA.

Abbreviations: LSK, Lin−Sca1+cKit+ cells (hematopoietic stem and progenitor cells); LT-HSC, long-term hematopoietic stem cells; ST-HSC, short-term hematopoietic stem cells; MPP2-4, multipotent progenitors 2-4; BM CBC, bone marrow complete cell count.

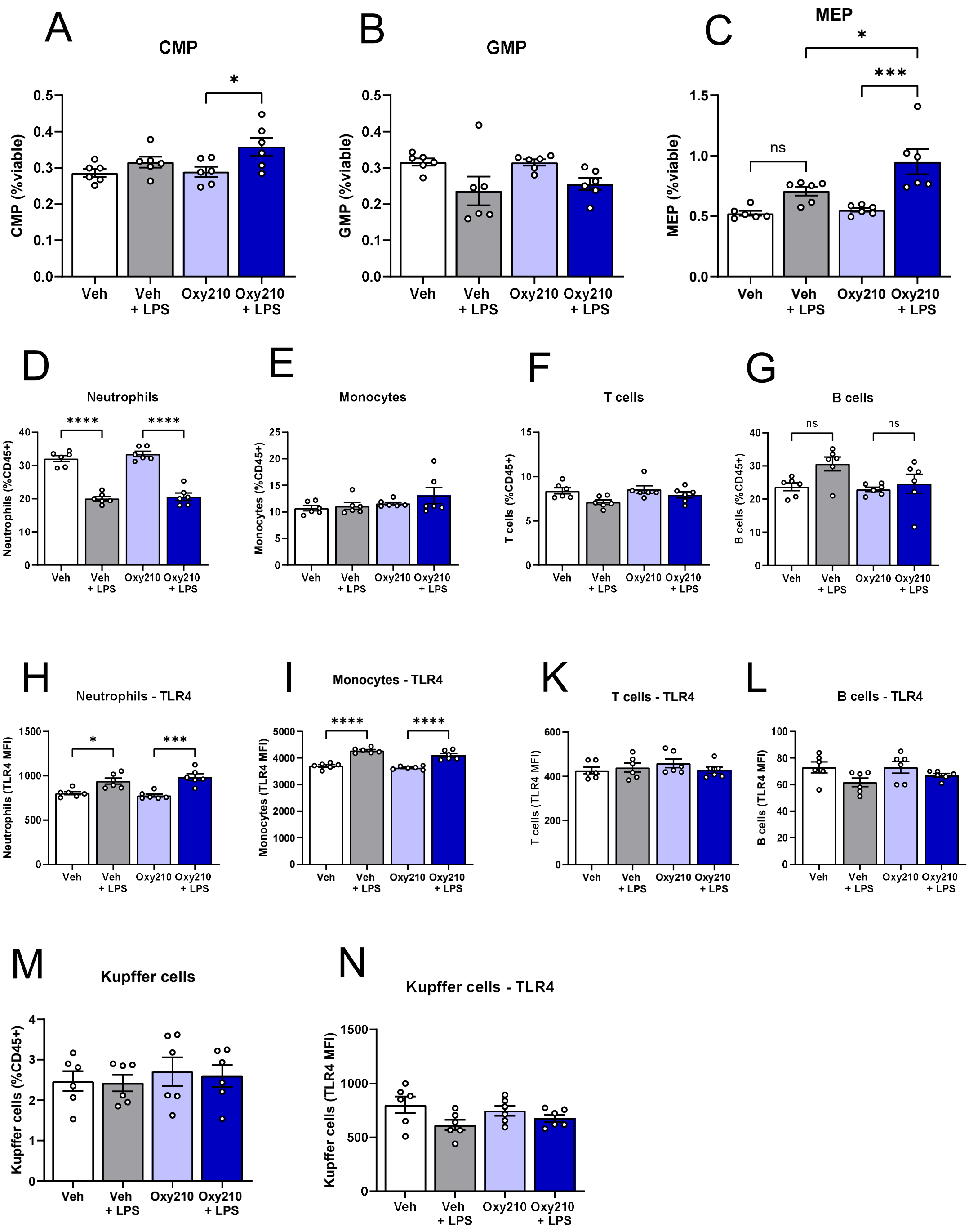
A
CMP
CMP (%viable)
0.0
0.1
0.2
0.3
0.4
0.5
*
Veh
Veh + LPS
Oxy210
Oxy210 + LPS
B
GMP
GMP (%viable)
0.0
0.1
0.2
0.3
0.4
0.5
Veh
Veh + LPS
Oxy210
Oxy210 + LPS
C
MEP
MEP (%viable)
0.0
0.5
1.0
1.5
ns
*
***
Veh
Veh + LPS
Oxy210
Oxy210 + LPS
D
Neutrophils
Neutrophils (%CD45+)
0
10
20
30
40
****
****
Veh
Veh + LPS
Oxy210
Oxy210 + LPS
E
Monocytes
Monocytes (%CD45+)
0
5
10
15
20
25
Veh
Veh + LPS
Oxy210
Oxy210 + LPS
F
T cells
T cells (%CD45+)
0
5
10
15
Veh
Veh + LPS
Oxy210
Oxy210 + LPS
G
B cells
B cells (%CD45+)
0
10
20
30
40
ns
ns
Veh
Veh + LPS
Oxy210
Oxy210 + LPS
H
Neutrophils - TLR4
Neutrophils (TLR4 MFI)
0
500
1000
1500
*
***
Veh
Veh + LPS
Oxy210
Oxy210 + LPS
I
Monocytes - TLR4
Monocytes (TLR4 MFI)
0
1000
2000
3000
4000
5000
****
****
Veh
Veh + LPS
Oxy210
Oxy210 + LPS
K
T cells - TLR4
T cells (TLR4 MFI)
0
200
400
600
Veh
Veh + LPS
Oxy210
Oxy210 + LPS
L
B cells - TLR4
B cells (TLR4 MFI)
0
20
40
60
80
100
Veh
Veh + LPS
Oxy210
Oxy210 + LPS
M
Kupffer cells
Kupffer cells (%CD45+)
0
1
2
3
4
Veh
Veh + LPS
Oxy210
Oxy210 + LPS
N
Kupffer cells - TLR4
Kupffer cells (TLR4 MFI)
0
500
1000
1500
Veh
Veh + LPS
Oxy210
Oxy210 + LPS

Figure S3. The effect of Oxy210 on bone marrow myeloid progenitor cells and leukocytes, and Kupffer cells

Wild-type mice were fed Oxy210 or vehicle (Veh) control for 4 days. On day 4, a subset of animals was challenged with LPS (5µg/mouse; i.p.), and an analysis of BM myeloid progenitors and leukocytes and liver Kupfer cells was performed 2 hours later.

A-C – Bone marrow myeloid progenitor cells

Cell frequency (% of viable cells) of **(A)** CMP (cKit^{+}CD16/32^{mid}CD34^{+}), **(B)** GMP (cKit^{+}CD16/32^{+}CD34^{+}), and **(C)** MEP (cKit^{+}CD16/32^{-}CD34^{-}) in the BM of mice (n=6 mice/group).

D-L - Bone marrow leukocytes

D-G. Cell frequency (% of CD45) of **(D)** neutrophils (CD45^{+}Gr1^{+}CD11b^{+}), **(E)** monocytes (CD45^{+}CD115^{+}Gr1^{+}CD11b^{+}), **(F)** T cells (CD45^{+}CD3^{+}CD11b^{-}) and **(G)** B cells (CD45^{+}B220^{+}CD11b^{-}) in the BM of mice (n=6 mice/group).

H-L. BM leukocytes were stained with TLR4 and surface levels were measured by flow cytometry. MFI of TLR4 staining is shown.

M,N - Kupffer cells

(M) Cell frequency (% of CD45), and **(N)** TLR4 surface staining on Kupffer cells CD45^{+}, CD11b^{int}, F4/80^{+}, CD64^{+}, MHCII^{+}, TIM4^{+}) from the liver of mice (n=6 mice/group).

Data are presented as mean ± SEM. Data points represent individual mice. Significance was determined by two-way ANOVA.

Abbreviations: CMP, common myeloid progenitor; GMP, granulocyte-monocyte progenitor; MEP, megakaryocyte-erythroid progenitor.

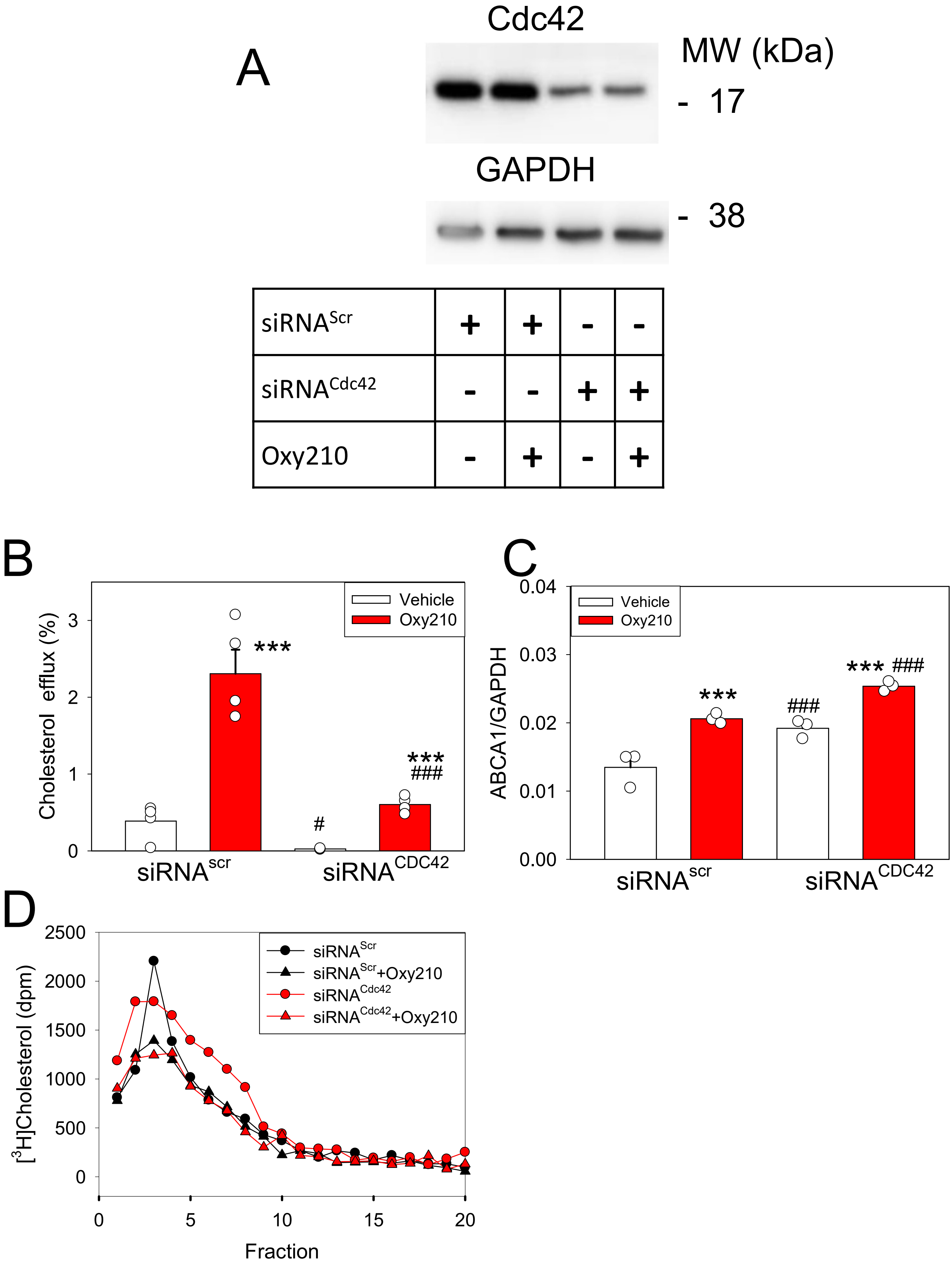

Figure S4. The effect of silencing of Cdc42 on ability of Oxy210 to reduce lipid rafts abundance in RAW264.7 cells.

A – The effect of Cdc42 silencing and of Oxy210 on Cdc42 abundance.

B - The effect of Cdc42 silencing and of Oxy210 on cholesterol efflux to apoA-I.

C – The effect of Cdc42 silencing and of Oxy210 on the abundance of total ABCA1.

D - The effect of Cdc42 silencing and of Oxy210 on lipid raft abundance. Distribution of [^{3}H]cholesterol in density gradient centrifugation fractions.

***$p<0.001$ *versus* vehicle; #$p<0.05$; $^{###}p<0.001$ *versus* siRNA^{scr} .

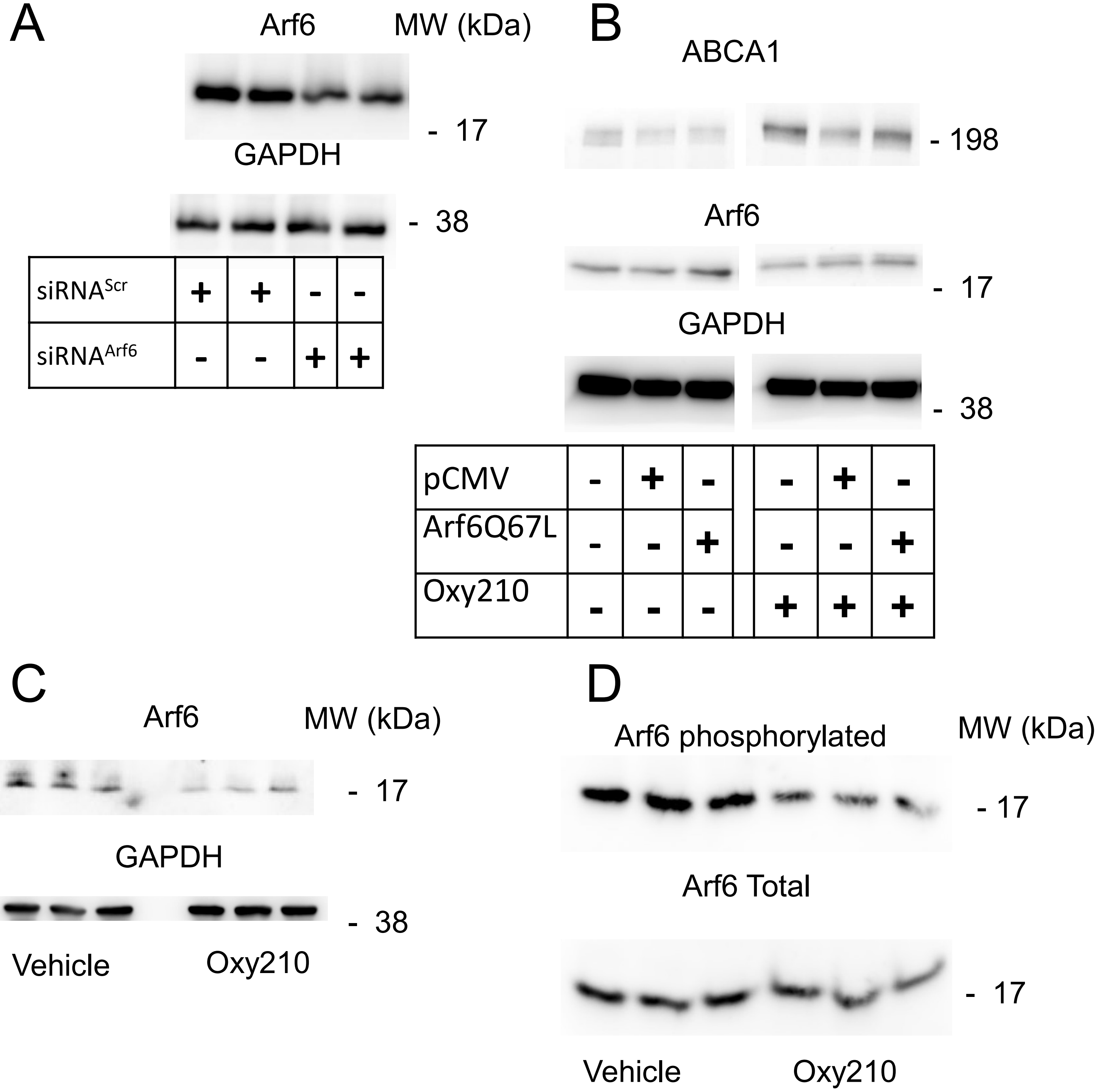

Figure S5. The effects of Oxy210 on ARF6

A - The effect of Arf6 silencing on Arf6 abundance.

B - The effect of Arf6 overexpression on the abundance of Arf6 and ABCA1

C – The effect of Oxy210 on Arf6 abundance

D – The effect of Oxy210 on Arf6 activation (phosphorylation)

Table S1. **Analysis of [^{3}H]cholesterol distribution along density gradient**

Cells	Vehicle AUC* (dpm x 10^3)	Oxy210 AUC* (dpm x 10^3)	p**
SupT1	14.6±0.6	10.7±1.3	0.027
RAW264.7	14.4±1.9	7.7±1.1	<0.001
BMDM	14.1±2.4	9.9±1.7	<0.001
SHSY5Y	17.7±1.6	12.6±1.1	0.001

*Area under the curve of [^{3}H]cholesterol distribution along density gradient ±SEM; ** t-test

Table S2. **The effect of Oxy210 on the abundance of lipid rafts in ABCA1$^{+/+}$ and ABCA1$^{-/-}$ human fibroblasts.**

Cells	Vehicle AUC* (dpm x 10^3)	Oxy210 AUC* (dpm x 10^3)	p**
**ABCA1$^{+/+}$**	4.6±0.8	3.2±0.4	0.04
**ABCA1$^{-/-}$**	6.1±0.7	2.2±0.3	<0.001

*Area under the curve of [^{3}H]cholesterol distribution along density gradient ±SEM;

** t-test.

Table S3. **The effect of Oxy210 on the abundance of lipid rafts in RAW264.1 cells with and without silencing of Cdc42.**

Cells	Vehicle AUC* (dpm x 10^3)	Oxy210 AUC* (dpm x 10^3)	p
**$siRNA^{scr}$**	8.1±1.4	6.9±0.8	0.05**
**$siRNA^{Cdc42}$**	9.4±1.0	7.6±1.0	<0.001***

*Area under the curve of [^{3}H]cholesterol distribution along density gradient ±SEM;

rank test; * t-test

Table S4. **The effect of Oxy210 on the abundance of lipid rafts in RAW264.1 cells loaded with plasmalogen.**

Loading	Vehicle AUC* (dpm x 10^3)	Oxy210 AUC* (dpm x 10^3)	p**
**Vehicle**	16.3±0.9	14.0±0.7	0.045
**Plasmalogen**	14.7±0.8	12.7±0.5	0.074

*Area under the curve of [^{3}H]cholesterol distribution along density gradient ±SEM;

* t-test

Table S5. **The effect of Oxy210 on the abundance of lipid rafts in RAW264.1 cells with and without silencing of Arf6**

Cells	Vehicle (AUC* (dpm x 10^3)	Oxy210 (AUC* (dpm x 10^3)	p**
**$siRNA^{scr}$**	11.9±0.5	6.887±0.8	<0.001
**$siRNA^{Arf6}$**	7.2±0.5	7.7±0.4	0.13

*Area under the curve of [^{3}H]cholesterol distribution along density gradient ±SEM;

** t-test

Table S6 **The effect of Oxy210 on the abundance of lipid rafts in RAW264.1 cells with and without overexpressing Arf6Q67L.**

Cells	Vehicle (AUC* (dpm x 10^3)	Oxy210 (AUC* (dpm x 10^3)	p (vehicle *vs* Oxy210)
**pCMV**	4.3±0.1	3.6±0.1	<0.05**
**Arf6Q67L**	4.3±0.1	6.8±0.2	<0.001**
**p (pCMV *vs* Arf6Q67L)**	0.47**	<0.001***	

*Area under the curve of [^{3}H]cholesterol distribution along density gradient ±SEM;

** t-test , *** two-way ANOVA
